# The Ethylene Biosynthesis Gene *CpACO1A*: A New Player in the Regulation of Sex Determination and Female Flower Development in *Cucurbita pepo*

**DOI:** 10.3389/fpls.2021.817922

**Published:** 2022-01-24

**Authors:** Gustavo Cebrián, Jessica Iglesias-Moya, Jonathan Romero, Cecilia Martínez, Dolores Garrido, Manuel Jamilena

**Affiliations:** ^1^Department of Biology and Geology, Agrifood Campus of International Excellence and Research Centre CIAMBITAL, University of Almería, Almería, Spain; ^2^Department of Plant Physiology, University of Granada, Granada, Spain

**Keywords:** *ACO* gene regulation, andromonoecy, monoecy, ethylene, flower maturation, parthenocarpy

## Abstract

A methanesulfonate-generated mutant has been identified in *Cucurbita pepo* that alters sex determination. The mutation converts female into hermaphrodite flowers and disrupts the growth rate and maturation of petals and carpels, delaying female flower opening, and promoting the growth rate of ovaries and the parthenocarpic development of the fruit. Whole-genome resequencing allowed identification of the causal mutation of the phenotypes as a missense mutation in the coding region of *CpACO1A*, which encodes for a type I ACO enzyme that shares a high identity with *Cucumis sativus* CsACO3 and *Cucumis melo* CmACO1. The so-called *aco1a* reduced ACO1 activity and ethylene production in the different organs where the gene is expressed, and reduced ethylene sensitivity in flowers. Other sex-determining genes, such as *CpACO2B*, *CpACS11A*, and *CpACS27A*, were differentially expressed in the mutant, indicating that ethylene provided by CpACO1A but also the transcriptional regulation of *CpACO1A*, *CpACO2B*, *CpACS11A*, and *CpACS27A* are responsible for determining the fate of the floral meristem toward a female flower, promoting the development of carpels and arresting the development of stamens. The positive regulation of ethylene on petal maturation and flower opening can be mediated by inducing the biosynthesis of JA, while its negative control on ovary growth and fruit set could be mediated by its repressive effect on IAA biosynthesis.

## Highlights

– CpACO1A is a type I ACO enzyme involved in ethylene production in different *Cucurbita pepo* organs.
– A mutation in *CpACO1A* disrupts ethylene production and converts female into hermaphrodite flowers.
– Transcription of the ethylene biosynthesis genes is feedback-regulated in the female flower.
– The mutation *aco1a* alters the homeostasis of IAA, ABA, JA, and SA in the female flower.

## Introduction

The cultivated species of the Cucurbitaceae family are a group of monoecious plants that have been utilized as a model for the study of the genetic control of sex determination in plants (Martínez and Jamilena, 2021). Many varieties in cultivated species are monoecious, developing male and female flower in the same plant, but some of the varieties are andromonoecious (male and hermaphroditic flowers), trimonoecious (male, female, and hermaphroditic flowers), gynoecious (only female flowers), and androecious (only male flowers). This natural variability makes this an ideal family to investigate the genetics of sex determination. The first sex-determining genes were discovered in *Cucumis sativus* (cucumber) and *Cucumis melo* (melon) (Boualem et al., 2008, 2009, 2015; Martin et al., 2009; Chen et al., 2016), but recent years have witnessed important discoveries in *Cucurbita pepo* (pumpkin and squash) (Martínez et al., 2014; García et al., 2020a,b) and *Citrullus lanatus* (watermelon) (Boualem et al., 2016; Ji et al., 2016; Manzano et al., 2016; Aguado et al., 2020; Zhang et al., 2020). Although many of the findings are similar in all species, the genetic control of sexual determination in some species differs slightly from the rest of the species (Aguado et al., 2020).

Ethylene is the key regulator of sex determination in cucurbits. External treatments with ethylene-releasing or -inhibiting agents have been used to determine the role of this hormone in the control of sex expression, i.e., female flowering transition and the number of female and male flowers per plant, as well as sex determination, which are the mechanisms that lead to a female or a male flower from a potentially hermaphrodite floral bud (Manzano et al., 2013, 2014). The latter was achieved by arresting the growth of the stamens or carpels, respectively (Bai et al., 2004). Ethylene increases the ratio of female to male flowers in *Cucumis* and *Cucurbita* (Rudich et al., 1969; Byers et al., 1972; Manzano et al., 2011, 2013), but reduces this ratio in *Citrullus* plants. Inhibition of ethylene biosynthesis or perception, on the other hand, reduces the number of female flowers per plant in *Cucumis* and *Cucurbita*, and transforms the female flowers into bisexual or hermaphrodite ones. In *Citrullus*, this last treatment increases the number of female flowers per plant, but also transforms female flowers into hermaphroditic flowers, indicating that ethylene is required to arrest the development of stamens in female flowers of all cucurbits (Manzano et al., 2014). Although gibberellins, auxins, and brassinosteroids have also been associated with sex control in cucurbits, some of their functions seem to be mediated by ethylene (Papadopoulou et al., 2005; Manzano et al., 2011; Zhang et al., 2014, 2017).

So far, all of the discovered sex-determining genes are either in the ethylene biosynthesis and signaling pathway, or are transcriptional factors that regulate the former. The gene that regulates abortion of stamens during the formation of a female flower in all studied cucurbits encodes for an ethylene biosynthesis enzyme: cucumber *ACS2* and its orthologs (Boualem et al., 2008, 2009, 2016; Martínez et al., 2014; Ji et al., 2016; Manzano et al., 2016). This female-forming gene is negatively regulated by the transcription factor *WIP1*, which is responsible for the arrest of carpels in the formation of male flowers (Martin et al., 2009; Hu et al., 2017; Zhang et al., 2020). The male-forming *WIP1* gene is negatively regulated by *ACS11* and *ACO2/ACO3* in cucumber and melon, which are expressed very early in the floral meristem and determine the formation of a female flower. The disruption of either of these genes promotes the conversion of monoecy into androecy (Boualem et al., 2015; Chen et al., 2016). EMS mutation in ethylene receptor genes of *C. pepo* has demonstrated that ethylene perception at early and late stages of flower development is crucial for female flower determination. The *etr1a*, *etr1a-1*, *etr1b*, and *etr2b* gain of function mutations, in fact, lead to andromonoecy and androecy concomitantly with a reduced ethylene sensitivity (García et al., 2018, 2020a,b).

The role of other ethylene biosynthesis genes in sex determination is unknown. In this paper, we demonstrate that the ethylene biosynthesis gene *CpACO1A* is involved in sex determination and flower development in *C. pepo*. Although the gene is not flower-specific, its role in ethylene biosynthesis is required for arresting stamen development, and the proper maturation and development of corolla and ovary of the female flower. *CpACO1A* and other sex-determining *ACO* and *ACS* ethylene biosynthesis genes were regulated by *CpACO1A*-producing ethylene in the female flower. The ethylene provided by CpACO1A also regulates hormonal balance in the female flowers. The increased indole-3-acetic acid (IAA) and the reduced abscisic acid (ABA) and jasmonic acid (JA) contents in the *aco1a* mutant may be responsible for the parthenocarpic fruit development and the delayed flower opening of the mutant female flower.

## Materials and Methods

### Plant Material and Isolation of Mutants

The *aco1a* mutant analyzed in this study was isolated from a high-throughput screening of *C. pepo* EMS collection (García et al., 2018). M2 plants from 600 lines were grown to maturity under standard greenhouse conditions, and alterations in reproductive developmental traits were evaluated. A mutant family was detected that produced hermaphrodite flowers, instead of female flowers. This mutant was selected for further characterization, and named *aco1a*. The monitoring of the development of the growth of the female floral organs, corolla, and ovary, as well as the degree of their stamen development detected in the flowers, showed similarity with the phenotype found for other families of mutants previously described and characterized by García et al. (2020a,b) in *C. pepo*, which led us to deduce the possible relationship of ethylene with this new mutation. Prior to phenotyping, *aco1a* mutant plants were crossed twice with the background genotype MUC16, and the resulting BC_2_ generation was selfed to obtain the BC_2_S_1_ generation.

### Phenotyping for Monoecy Stability, Sex Expression, and Floral Traits

The total of 300 BC_2_S_1_ plants from wt/wt, wt*/aco1a*, and *aco1a/aco1a* were transplanted to a greenhouse and grown to maturity under local greenhouse conditions without climate control, and under standard crop management of the region, in Almería, Spain. The sex phenotype of each plant was determined according to the sex of the flowers in the first 40 nodes of each plant. A minimum of 30 wt/wt, 30 wt*/aco1a*, and 30 *aco1a/aco1a* plants were phenotyped. Phenotypic evaluations were performed in the spring-summer seasons 2019 and 2020.

The sex expression of each genotype was assessed by determining the node at which plants transitioned to pistillate flowering, and the number of male or pistillate/hermaphrodite flower nodes. The sex phenotype of each individual pistillate/hermaphrodite flower was assessed by the so-called andromonoecy index (AI) (Martínez et al., 2014; Manzano et al., 2016). Pistillate flowers were separated into three phenotypic classes that were given a score from 0 to 3 according to the degree of their stamen development: female (AI = 0), showing no stamen development; bisexual or pistillate (AI = 1; AI = 2), showing partial development of stamens and no pollen; and hermaphrodite (AI = 3), showing complete development of stamens and pollen. The average AI of each plant and each genotype was then assessed from the resulting AI score of at least 10 individual female flowers from each plant, using a minimum of 30 plants for each genotype. To assess floral organ development, the growth rates of ovaries and petals in both female and male flowers of WT and *aco1a* mutant plants were determined by measuring the length and diameter of these floral organs every 2 day for 28 day in 20 flowers of each genotype, starting with flower buds ∼2 mm in length. The anthesis time was estimated as the number of days taken for a 2 mm pistillate or male floral bud to reach anthesis.

### Identification of *aco1a* Mutation by Whole-Genome Sequencing Analysis

To identify the causal mutations of the *aco1a* phenotype, WT, and mutant plants derived from BC_1_S_1_-segregating populations were subjected to whole-genome sequencing (WGS). In total, 120 BC_1_S_1_ seedlings were transplanted to a greenhouse and grown to maturity. The phenotype of those seedlings was verified in the adult plants, as wt/wt and wt/*aco1a* plants were monoecious while *aco1a/aco1a* plants were andromonoecious or partially andromonoecious.

The genomic DNA from 30 WT and 30 *aco1a* plants was isolated by using the Gene JET Genomic DNA Purification Kit (Thermo Fisher Scientific ^®^), and pooled into two different bulks: WT bulk and *aco1a* mutant bulk. DNA from each bulk was randomly sheared into short fragments of approximately 350 bp for library construction using the NEBNext ^®^ DNA Library Prep Kit^1^, and fragments were briefly PCR enriched with indexed oligos. Pair-end sequencing was performed using the Illumina ^®^ sequencing platform, with a read length of PE150 bp at each end. The effective sequencing data were aligned with the reference *C. pepo* genome v.4.1 through BWA software (Li and Durbin, 2009). Single nucleotide polymorphisms (SNPs) were detected using the GATK HAPLOTYPECALLER (Depristo et al., 2011). ANNOVAR was used to annotate the detected SNPs (Wang et al., 2010). Common variants between these mutant families (and other sequenced mutant families in the laboratory) were discarded, as they are likely common genomic differences with the reference genome. The genotype of the WT bulk (wt/wt and wt/*aco1a* plants) was expected to be 0/1 with an alternative allelic frequency (AF) of 0.3, while the genotype of the mutant bulk (*aco1a/aco1a* plants) was expected to be 1/1 with an AF = 1. Therefore, the sequencing data were filtered according to the following parameters: genotype quality ≥ 90, read depth ≥ 10, AF = 1 in the mutant bulks, and AF ≤ 0.3 in the WT bulks. Once we had a set of positions that were differentially enriched in each bulk, we filtered out SNPs that were not canonical EMS changes (G > A or C > T transitions) (Till et al., 2004). All filters were performed with RStudio ^®^ software. The impact of this final set EMS SNPs on gene function was finally determined by using Integrative Genomics Viewer (IGV) software and the Cucurbit Genomics Database (CuGenDB)^2^.

### Validation of the Identified Mutations by High-Throughput Genotyping of Individual Segregating Plants

Segregation analysis was performed to confirm that the identified mutations were causal mutations of the *aco1a* phenotype. Approximately 300 BC_2_S_1_ plants segregating for the mutation were genotyped using Kompetitive allele-specific PCR (KASP) technology. Primers were synthesized by LGC Genomics ®^3^, and the KASP assay was performed in the FX96 Touch Real−Time PCR Detection System (Bio-Rad ^®^) using the LGC protocol^4^. The multiplex PCRs were run with 10 μL final reaction volume containing 5 μL KASP V4.0 2× Master mix standard ROX (LCG Genomics ^®^), 0.14 μL KASP-by-Design primer mix (LCG Genomics ^®^), 2 μL of 10–20 ng/μL genomic DNA, and 2.86 μL of water. The PCR thermocycling conditions were 15 min at 94°C (hot-start activation) followed by 10 cycles of 94°C for 20 s and 61°C for 1 min (dropping −0.6°C per cycle to achieve a 55°C annealing temperature) followed by 26 cycles of 94°C for 20 s and 55°C for 1 min. Data were then analyzed using CFX Maestro™ Software (Bio-Rad ^®^) to identify SNP genotypes.

### 1-Aminocyclopropane-1-Carboxylic Acid Oxidase Enzyme Activity

1-Aminocyclopropane-1-carboxylic acid oxidase (ACO) activity was assessed following the protocol described in Bulens et al. (2011). The enzyme activity was quantified in leaves, stems, roots, cotyledon, and flowers in triplicate. About 0.5 g of each material was pulverized in liquid nitrogen, and 1 mL of extraction buffer MOPS (pH 7.2) and 50 mg of polyvinylpolypyrrolidone (PVPP) were added to each sample. The samples were subsequently incubated for 10 min at 4°C and finally centrifuged for 30 min at 22,000 × *g* at 4°C. About 400 μL of the resulting supernatant was mixed with 3.6 mL of MOPS reaction buffer (pH 7.2) in a 20 mL glass vial. After homogenizing the mixture for 5 s, samples were incubated in a water bath for 1 h at 30°C while gently shaking. The amount of ethylene formed was determined by analyzing 1 mL of gas from the headspace of the reaction tube on a Varian ^®^ 3900 gas chromatograph (GC) fitted with a flame ionisation detector (FID). A blank sample (3.6 mL reaction buffer + 400 μL DW) was used as a control for the whole process. Enzyme reactions and ethylene readings were done in triplicate. The activity of ACO was expressed as nmol × gFW^–1^ × h^–1^.

### Ethylene Production Measurements

The production of ethylene in WT and *aco1a* flowers was assessed throughout the different stages of development. Female and male floral buds of 8–55 mm in length (FFB/MFB) and the apical shoots of plants growing under climatic controlled conditions were collected and incubated at room temperature for 6 h in hermetic glass containers of 50–450 mL. Ethylene production was determined by analyzing 1 mL of gas from the headspace in a Varian ^®^ 3900 gas chromatograph (GC) fitted with a FID. The instrument was calibrated with standard ethylene gas. Four biological replicates were made for each one of the flower developmental stages analyzed and three measurements per sample. Ethylene production was expressed as nL × gFW^–1^ × 6 h^–1^.

### Assessing Ethylene Sensitivity

To evaluate the level of sensitivity to ethylene, flower abscission was assessed for male flowers in response to an external treatment with ethylene. Male flowers from WT and *aco1a* plants were collected at two stages of development: A (anthesis) and A-2 (2 day before anthesis). For each stage, 30 WT and *aco1a* flowers were placed in glass vases with water, and incubated in two culture chambers with equal humidity and temperature, 50% RH and 20°C. One of the chambers was used as a control (Ct), and the other was filled with 50 ppm of ethylene (ET). The tests were performed in triplicate. Both chambers remained closed for 72 h, and the percentage of abscission produced was evaluated after 24, 36, 48, and 72 h for each stage of development.

### Hormone Concentration Measurements

Female flower buds of 5–8 mm from WT and *aco1a* plants were collected for hormone concentration measurements. Representative samples consisted of three bulks of approximately 30 female flowers each. To preserve the samples, WT and *aco1a* bulks were quickly stored on dry ice. Then, the samples were placed in a freeze dryer CRYODOS V3.1-50 (Telstar ^®^), where they were lyophilized for 1 week and subsequently pulverized in a mixer mill MM200 (Retsch™). The concentration of salicylic acid (SA), indol-3-butyric acid (IBA), IAA, gibberellic acid (GA3), 6-benzyladenine (BA), ABA, and JA were determined in each triplicated sample through ultra-performance liquid chromatography coupled with a hybrid quadrupole orthogonal time-of-flight mass spectrometer (UPLC-Q-TOF/MS/MS) according to the hormone determination method of Müller and Munné-Bosch (2011).

### Bioinformatics and Phylogenetic Analysis

Alignments and protein sequences analysis were performed using the BLAST alignment tools at NCBI^5^. Protein structure information and homology-modeling were analyzed using the Protein Data Bank RCSB PDB^6^ and SWISS-MODEL^7^. The phylogenetic relationships between *C. pepo*, *Arabidopsis thaliana*, *Solanum lycopersicum*, *Oryza sativa*, *C. sativus*, and *C. melo* of *ACO* genes were studied using MEGA X software (Kumar et al., 2018), which allowed the alignment of proteins and the construction of phylogenetic trees using MUSCLE (Edgar, 2004) and the maximum likelihood method based on the Poisson correction model (Zuckerkandl and Pauling, 1965), with 2,000 bootstrap replicates. The protein sequences (Supplementary Table 1) were obtained using the Arabidopsis Information Resource^8^, the Cucurbit Genomics Database (CuGenDB)^9^, the Rice Database Oryzabase-SHIGEN^10^, and the Sol Genomics Network^11^. Cucurbits *ACO* genes structure visualization, such as the composition and position of exons and introns, were performed with the Gene Structure Display Server (GSDS)^12^. Finally, Delta Delta G (ΔΔG), a metric for predicting how a single point mutation will affect protein stability, was assessed with the tools SNAP^213^ and I-Mutant3.0.^14^ MUpro^15^ and CUPSAT^16^ tools were also used to predict the stability of the CpACO1A protein.

### Assessment of Relative Gene Expression by Quantitative RT-PCR

Gene expression analysis was carried out in samples of WT and *aco1a* plants growing in a greenhouse during the spring-summer season. The expression level was studied in male and female flowers’ organs (corolla and ovaries) at different flower developmental stages, as well as in plant apical shoots, leaves, shoots, cotyledons, and roots. The analysis was performed in three biological replicates for each genotype, each of which was derived from a pool of four plants. Total RNA was isolated according to the protocol of the GeneJET Plant RNA Purification Kit (Thermo Fisher Scientific ^®^). RNA was converted into cDNA with the ADNc RevertAid™ kit (Thermo Fisher Scientific ^®^). The qRT-PCR was performed in 10 μL total volume with 1× Top Green qPCR Super Mix (Bio-Rad ^®^) in the CFX96 Touch Real-Time PCR Detection System thermocycler (Bio-Rad ^®^). The gene expression values were calculated using the 2^–ΔΔCT^ method (Livak and Schmittgen, 2001). The constitutive *EF1*α gene was used as the internal reference. Supplementary Table 2 shows the primers used for each qRT-PCR reaction.

### Statistical Analyses

Data were analyzed for multiple comparisons by analysis of variance (ANOVA) using the statistical software Statgraphic Centurion XVIII. Differences between genotypes and treatments were separated by least significant difference (LSD) at a significance level of *p* ≤ 0.05.

## Results

### *aco1a* Impairs Sex Determination and Petals and Ovary Development

The mutant *aco1a* was found in a high-throughput screening of a *C. pepo* mutant collection for alterations in flower and fruit development. To ensure accurate phenotyping, mutant plants were backcrossed with the background genotype MUC16 for two generations, and then selfed. The resulting BC_2_S_1_ generations segregated 3:1 for WT and *aco1a* phenotypes, indicating that the mutation is recessive (Supplementary Table 3).

**FIGURE 1:**
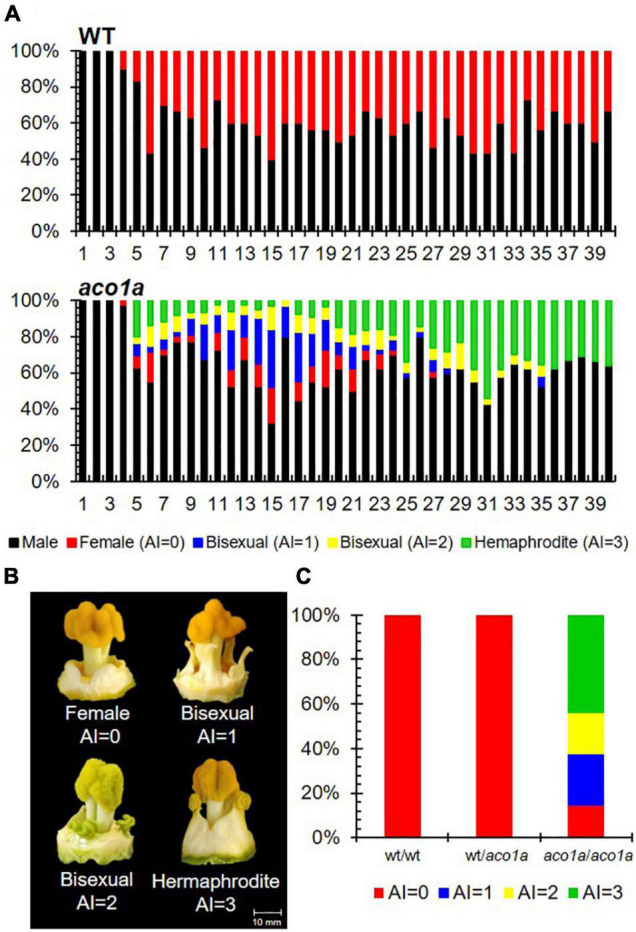
Sex phenotype of WT and *aco1a* plants. **(A)** Distribution of male and pistillate flowers in the first 40 nodes of the main shoot. In each node, color bars indicate the percentages of male (black), female (red), and bisexual and hermaphrodite flowers (blue, yellow, and green) in the total number of plants analyzed (*n* = 30 for each genotype). **(B)** Phenotype of pistillate flowers with different stamen development and AI index. Female flowers (AI = 0) develop no stamen, bisexual flowers (AI = 1–2) develop intermediate stamens, and hermaphrodite flowers (AI = 3) develop entire stamens with pollen. **(C)** Percentage of each pistillate flower in each genotype.

The sex phenotype of *aco1a* was assessed in BC_2_S_1_ plants growing under spring-summer conditions (Figure 1). Male flowers were not affected, but most female flowers were converted into bisexual flowers with partially or totally developed stamens (Figure 1A). This partial conversion of monoecy into andromonoecy, also termed unstable monoecy, partial andromonoecy, or trimonoecy, indicates that *aco1a* impairs the sex determination mechanism which is responsible for arresting stamen development in the female flower. Pistillate flowers in the first 40 nodes were classified according to the andromonoecious index (AI) in either homozygous WT (wt/wt), heterozygous (wt*/aco1a*), or homozygous mutant (*aco1a/aco1a*) plants (Figure 1B). The wt/wt and wt*/aco1a* plants produced only female flowers (AI = 0), indicating a complete arrest of stamen development in the pistillate flowers of these plants (Figure 1C). The *aco1a/aco1a* pistillate flowers, however, exhibited different degrees of stamen development (AI ranging from 0 to 3), and plants had an average AI of 2.1 (Figures 1B,C).

**FIGURE 2:**
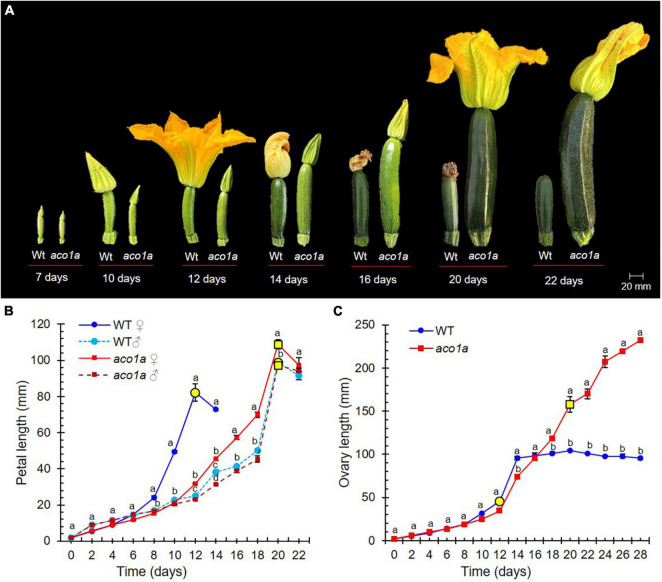
Comparison of WT and *aco1a* flower development. **(A)** Effect of *aco1a* mutation on the development of ovary and corolla of the pistillate flower. Note that the mutant pistillate flower reaches anthesis later than the WT, and that the ovary continues its growth until producing a parthenocarpic fruit. **(B)** Comparison of the growth rate of WT and mutant corolla. Flowers were labeled when their ovaries were 2 mm long, and then measured every 2 days for 22 days. Yellow circles indicate the time at which more than 80% of the flowers reached anthesis. **(C)** Comparison of the growth rate of WT and mutant ovaries/fruits over a period of 22 days. Error bars represent SE. Different letters indicate significant differences in flowers of the different genotypes at each developmental time (*p* ≤ 0.05).

Figure 2 shows the effects of the *aco1a* mutation on petal and ovary/fruit development. In the bisexual and hermaphrodite flowers of *aco1a* (AI = 2–3), the petal growth rate was reduced and resembled petal development in male flowers. Petal maturity and subsequent anthesis of the flower were delayed in the mutant with respect to WT (Figures 2A,B). Anthesis time, the period of time taken for a 2 mm floral bud to reach anthesis and to open, was longer in male WT flowers (average 21 days) than in female WT flowers (average 12 days) (Figure 2B). Bisexual and hermaphrodite *aco1a* flowers also took an average of 21 days to reach anthesis (range 20–25 days). No alterations in petal development or anthesis time were observed in WT and *aco1a* male flowers (Figure 2B).

Pollination was attempted in *aco1a* hermaphrodite flowers (AI = 2–3), but none of the fruits were able to set seeds. Since the pollen is fertile in other plants, this female sterility could be associated with the over-maturation of stigma and style because of the delayed corolla aperture. However, we were able to self *aco1a/aco1a* plants by using the few female flowers with no stamen (AI = 0). Significant differences were detected in ovary size between WT and *aco1a* pistillate flowers (Figures 2A,C). At anthesis, the WT ovary reached approximately 12 cm in length and then aborted. The *aco1a* ovary, in contrast, continued to grow until it reached 18–30 cm at anthesis (Figures 2A,C). The growth rate of WT and *aco1a* ovary/fruit was similar during the first 16 days. After that time, WT ovaries aborted, and those of *aco1a* maintained growth up to anthesis (Figure 2C). The *aco1a* fruits can be considered parthenocarpic since they grew in the absence of pollination, as the corolla was closed.

### *aco1a* Is a Missense Mutation Causing P5L Substitution in the Ethylene Biosynthesis Enzyme CpACO1A

To elucidate the causal mutation of *aco1a* phenotype, we performed WGS of two bulked DNA samples from a BC_2_S_1_ segregating population: the WT bulk, having DNA from 30 WT plants (monoecious); and the *aco1a* bulk, having DNA from 30 mutant plants (partially andromonoecious). In the mutant bulk, only the plants that showed the most extreme andromonoecious phenotype were selected.

**FIGURE 3:**
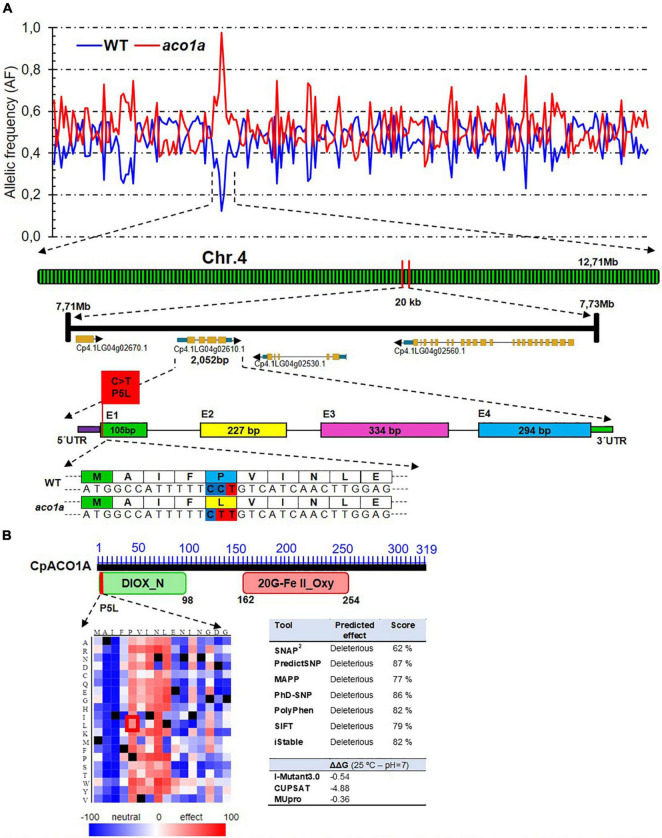
Identification of *aco1a* causal mutation by BSA-sequencing. **(A)** Frequency of the alternate allele in the WT and *aco1a* bulks along the physical map of squash genome. The analysis indicates that a region of chromosome 4 is responsible for the mutant phenotype. A missense mutation on the gene *Cp4.1LG04g02610.1* (*CpACO1A*) produces a change of proline by leucine at residue 5 (P5L) of the ethylene biosynthesis enzyme CpACO1A. **(B)** Impact of P5L mutation in the N-terminal conservative non-heme dioxygenase DIOX_N region of ACO enzymes. The SNAP^2^-generated heatmap of CpACO1A N-domain indicates that changes in residue 5–9 have a high impact on protein function. The red box indicates the impact of the *aco1a* mutation. The table on the right shows the predicted effect of the mutation on protein function and stability by using different bioinformatics tools.

More than 98% of the sequencing reads (more than 80 million in each bulk) were mapped against the *C. pepo* reference genome version 4.1, which represented an average depth of 47.41 (Table 1). The identified SNPs (more than 370,000 in each of the bulks) were filtered for their mutant allele frequency (AF) in the WT and the mutant DNA bulks. For the causal mutation of the phenotype, it is expected that the genotype was 0/1 for WT bulk (alternative allele frequency AF = 0.25) and 1/1 for the mutant bulk (AF = 1). For the non-causal SNPs, however, we expected an AF of 0.5 in both bulks. A putative causal region in chromosome 4 was found that has the expected AF in WT and mutant bulk (Figure 3A). In fact, after filtering for AF = 1 in the mutant bulk and AF < 0.3 in the WT bulk, 412 SNPs were selected (Table 1). Among them, 145 corresponded to canonical EMS mutations (C > T and G > A), and only one on chromosome 4 was positioned on the exome and had a high impact on the protein (Table 1 and Figure 3).

**TABLE 1:** Summary sequencing data for WT and *aco1a*.

Sequencing	WT	*aco1a*
No. reads	106,448,438	84,600,742
Mapped reads (%)	98.10	98.06
Average depth	47.41	40.41
Coverage at least 4× (%)	95.81	95.41
**SNPs filtering**		
Total No. SNPs	381,666	374,917
AF (WT) < 0.3; AF (*aco1a*) = 1	412	412
EMS SNPs G > A or C > T	145	145
EMS SNPs (GQ > 90; DP > 10)	4	4
High impact SNPs	0	1

**Candidate SNP**		

**Chr**	**Position**	**Ref**	**Alt**	**Gene ID**	**Effect**	**Functional annotation**

4	7,715,975	C	T	Cp4.1LG04g02610	P5L	1-Aminocyclopropane-1-carboxylate oxidase 1

*AF, allelic frequency; GQ, genotype quality; DP, read depth.*

The sequence surrounding the candidate *aco1a* mutation (±500 bp) was then used in BLAST searches against the DNA and protein databases at NCBI. It was found that the C > T transition was a missense mutation changing proline by leucine at residue 5 (P5L) of the ethylene biosynthesis enzyme 1-aminocyclopropane-1-1carboxylate oxidase 1A (CpACO1A) (Figure 3A). To prove that the selected EMS mutation was the one responsible for *aco1a* phenotype, we genotyped the SNP alleles in 300 plants from a BC_2_S_1_ population. The results demonstrated a 100% co-segregation between the *aco1a* phenotype and the C > T mutation in *CpACO1A* (Supplementary Table 4). Other three EMS-induced mutations in chromosome 4 were also tested (Supplementary Table 5), but none of them co-segregated with the mutant phenotype in the 300 BC_2_S_1_ plants analyzed.

The identified mutation has a deleterious effect on CpACO1A enzyme (Figure 3B). Bioinformatics analysis with the SNAP^2^ tool predicted a negative effect of P5L substitution on protein function (Figure 3B). Other bioinformatics tools, such as PredictSNP, MAPP and iStable, among others, showed the same evidence (Figure 3B). Moreover, predicted Gibbs free energy changes (ΔΔG), a metric for predicting how a single point mutation could affect protein stability, was assessed for P5L mutation by using I-mutant3.0 predictor, CUPSAT, and MUpro. The comparison of *aco1a* and WT CpACO1A resulted in negative ΔΔG values, which indicated a decreased stability of the mutated protein (Figure 3B).

### Gene Structure and Phylogenetic Relationships of *CpACO1A*

**FIGURE 4:**
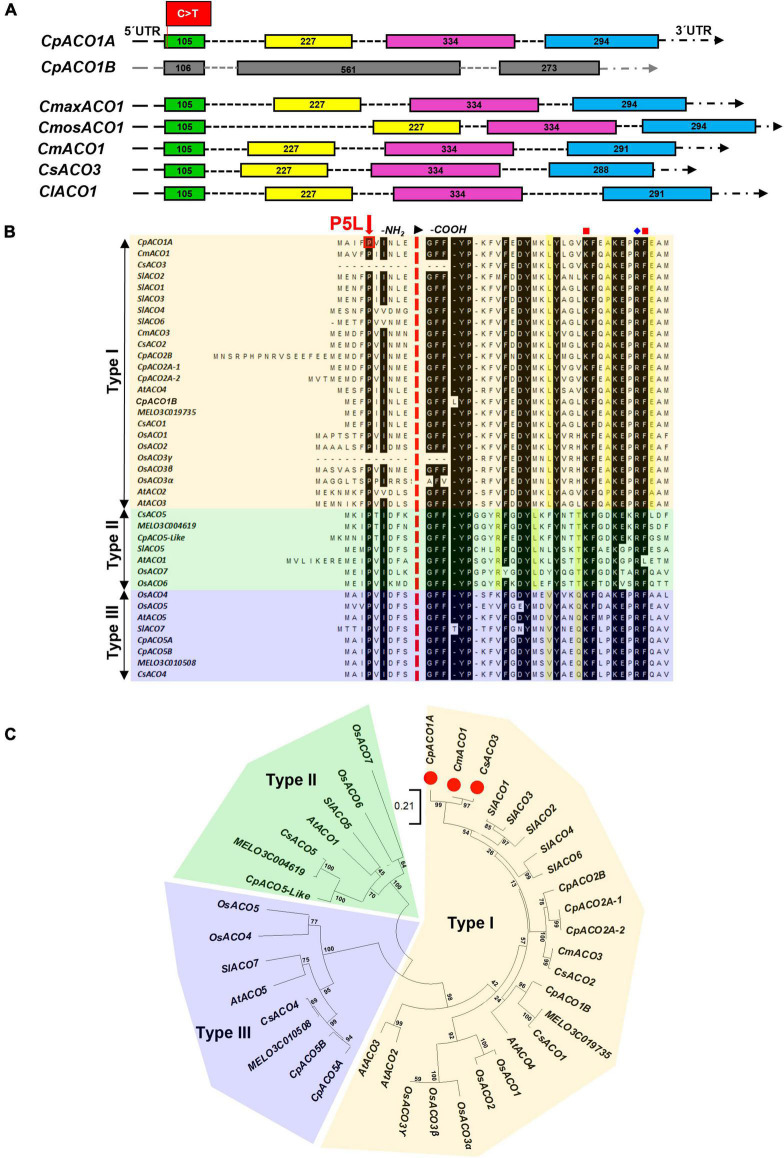
Genetic relationships among ACO enzymes in different plant species. **(A)** Comparison of the gene structure of *CpACO1A* and *CpACO1B* of *C. pepo* with other homologs in cucurbit species: *Cucurbita maxima* (*CmaxACO1*), *Cucurbita moschata* (*CmosACO1*), *Cucumis melo* (*CmACO1*), *Cucumis sativus* (*CsACO3*), and *Citrullus lanatus* (*ClACO1*). **(B)** Consensus proline residue in position 5 and ACO classification according to conserved residues in a specific position toward the -COOH end of the proteins. **(C)** Phylogenetic ACO tree from different cucurbit species: *C. melo*, *C. sativus*, and *C. pepo* together with those of the most studied model species, *Arabidopsis thaliana*, *Solanum lycopersicum*, and *Oryza sativa*. Bootstrap values for the main branches are depicted on the tree.

Given that the genomes of *C. pepo* are duplicated (Sun et al., 2017; Montero-Pau et al., 2018), the gene *CpACO1A* on chromosome 4 (Cp4.1LG04g02610) has a paralog (*CpACO1B*) with more than 80% of homology on an syntenic block of chromosome 5 (Cp4.1LG05g15190). The duplicates did not maintain the same molecular structure: four exons for *CpACO1A* and three exons for *CpACO1B* (Figure 4A). *ACO1*, like genes in other plants, including those of *Cucurbita maxima*, *Cucurbita moschata*, *C. melo*, *C. lanatus*, and *C. sativus*, conserve the four exonic structure of *CpACO1A* (Figure 4A). All ACO proteins in the NCBI database were found to conserve the proline residue on position five, indicating that this is an essential residue for ACO activity (Figure 4B).

Based on residues conserved at specific positions toward the carboxylic end of the proteins, three types of ACO enzymes have been established in plants (Figure 4B), which also defines its specific functionality and biological activity. A phylogenetic tree was inferred by using ACO protein sequences from different cucurbit species, including different *Cucurbita* sp., *C. melo* and *C. sativus*, together with those of the most studied model species, *A. thaliana*, *S. lycopersicum*, and *O. sativa* (Figures 4B,C). The *C. pepo* CpACO1A is a type I ACO that clustered together with melon CmACO1 and cucumber CsACO3 (also called CsACO1-like). Furthermore, the genes coding for the type I ACO1 enzymes of these three cucurbits were found to be positioned in a syntenic block of *C. pepo*, *C. melo*, and *C. sativus* genomes. The paralogous CpACO1B is also a type I ACO, but clustered separately from CpACO1A, CsACO3, and CmACO1 (Figure 4C).

### The *aco1a* Mutation Impairs *CpACO1A* Expression, ACO Activity, and Ethylene Production and Sensitivity

**FIGURE 5:**
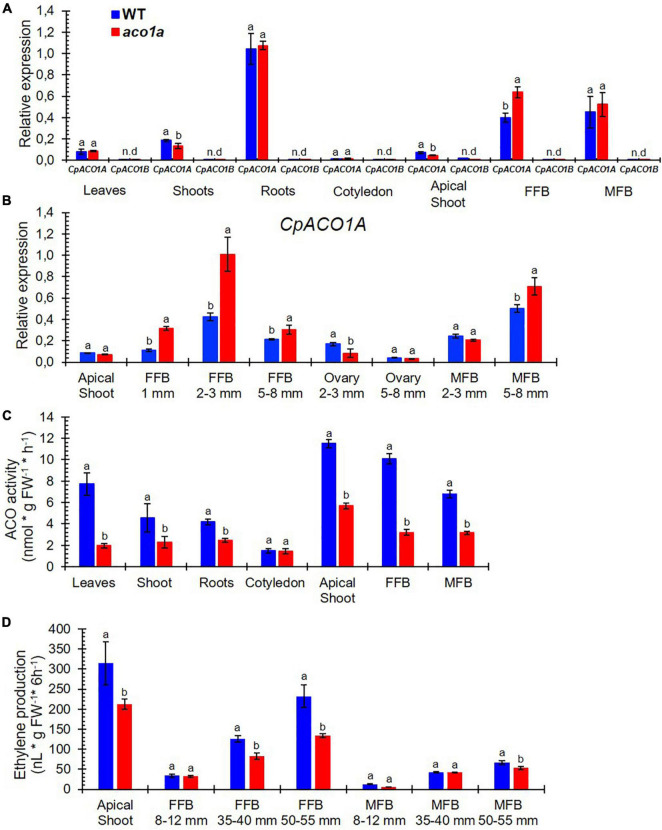
Comparison of *CpACO1A* gene expression, ACO activity, and ethylene production in WT and *aco1a* plants. **(A)** Relative gene expression of *CpACO1A* and *CpACO1B* in different WT and *aco1a* plant organs. **(B)** Relative expression of *CpACO1A* in the apical shoots and in female and male floral buds at different stages of development. **(C)** ACO1 activity in different WT and *aco1a plant* organs. **(D)** Ethylene production in the apical shoot and in female and male floral buds of WT and *aco1a* plants. FFB, female floral bud excluding the ovary; MFB, male floral bud. The assessments were performed in three independent replicates for each tissue. Error bars represent SE. Different letters indicate significant differences between WT and mutant organs at the same stage of development (*p* ≤ 0.05).

The expression of *CpACO1A* and *CpACO1B* in different WT and *aco1a* tissues is shown in Figure 5A. *CpACO1B* was not expressed in any of the analyzed tissues, indicating that this is a non-functional paralogous gene. *CpACO1A* was found to be expressed in all tissues, except in cotyledons. Its transcript was, however, much more accumulated in roots and flowers (Figure 5A). *CpACO1A* was similarly expressed in the different WT and mutant tissues, except in the female flower buds, where the gene showed a higher expression in the mutant. To understand the function of *CpACO1A* in flower development, its expression was compared in WT and *aco1a* apical shoots and female and male flowers buds (FFB/MFB) at different stages of development (Figure 5B). In the apical shoot, *CpACO1A* expression was similar in WT and *aco1a* plants. In the mutant female and male flowers, *CpACO1A* transcripts are similarly more highly accumulated in the mutants, suggesting that the andromonoecious *aco1a* phenotype is not caused by a reduction of gene expression.

Although there are other ACO isoenzymes in the *C. pepo* genome, we have assessed the total ACO activity and ethylene production in different WT and *aco1*a plant tissues (Figure 5C). The *aco1a* mutation was found to cause a reduction of ACO activity in all studied tissues, except in cotyledons, where the gene was not found to be expressed (Figure 5C). These data suggest that the mutation *aco1a* likely impairs CpACO1A activity.

The *aco1a* mutation significantly reduced the production of ethylene in the apical shoots of the plants, where a number of small floral buds are developing, and in pistillate flowers (Figure 5D). As previously reported, ethylene increased throughout the development of male, female (WT) and bisexual/hermaphrodite (*aco1a*) flowers, and pistillate flowers produced significantly more ethylene than male flowers at the same developmental stage (Figure 5D). The bisexual flowers of *aco1a* showed a significant reduction of ethylene production during their development, especially those flowers with more than 35 mm in length (Figure 5D). A slight reduction in ethylene production was also found in *aco1a* male flowers of 50–55 mm in length (Figure 5D).

**FIGURE 6:**
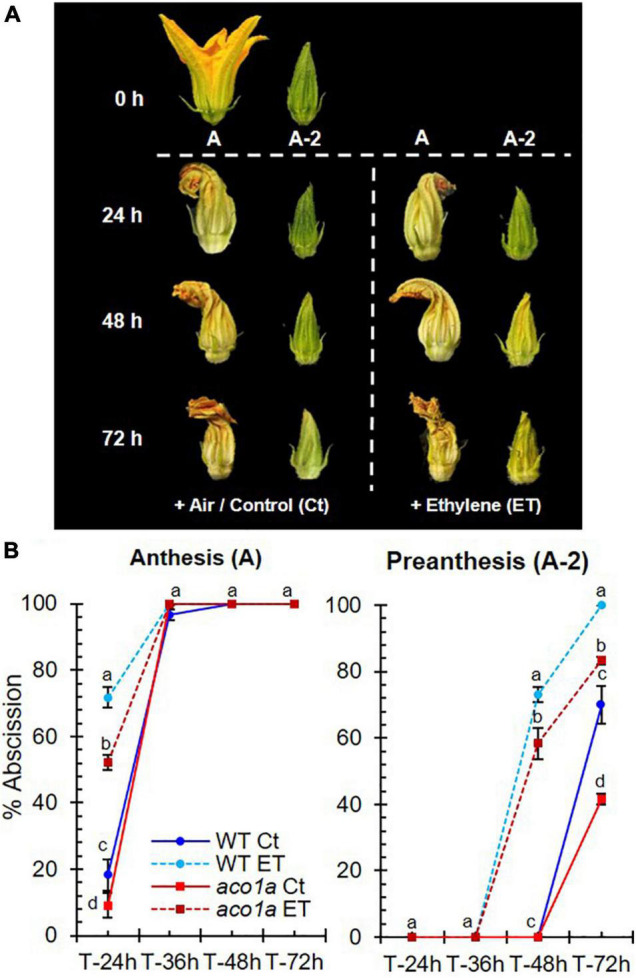
Ethylene sensitivity of WT and *aco1a* plants. The sensitivity to ethylene was determined by assessing the percentage of flower abscission in response to ethylene in male flowers at two stages of development. **(A)** Phenotype of male flowers at two stages of development: anthesis **(A)** and 2 days before anthesis (A-2). Photographs were taken at harvest (0 h of treatment) and 24, 48, and 72 h after the treatment with air or ethylene (ET). **(B)** Percentage of abscission in male flowers harvested at anthesis **(A)** or 2 days before anthesis (A-2). Error bars represent SE. Different letters indicate significant differences between flower abscission of each treatment and genotype at the same time after the treatment (*p* ≤ 0.05).

Ethylene sensitivity in WT and *aco1a* plants was also assessed by measuring the abscission time of male flowers in response to external treatments with ethylene (Figure 6). The male floral buds were collected at two developmental stages: anthesis (A) and 2 day before anthesis (A-2). The flowers were put in a container with water and treated in an atmosphere with air (control) or ethylene (ET) up to 72 h, and floral abscission was evaluated every 12 h (Figure 6A). Both WT and *aco1a* flowers responded to ethylene by accelerating their senescence and abscission (Figure 6B). However, the increase in the percentage of flower abscission in response to ethylene was lower in *aco1a* than in WT flowers (Figure 6B), indicating a partially ethylene-insensitive phenotype of the mutant *aco1a* male flowers.

### Expression of Different Sex-Determining Genes in WT and *aco1a* Flowers

**FIGURE 7:**
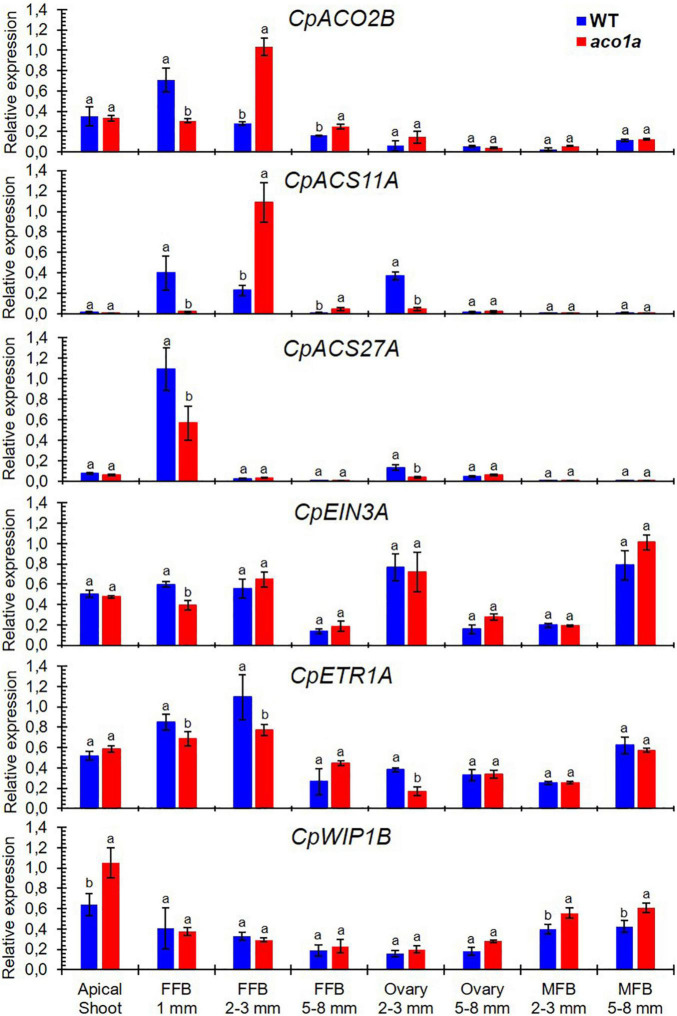
Relative expression of different sex-determining genes in the apical shoots and flowers of WT and *aco1a* plants. The expression was assessed for genes involved in ethylene biosynthesis (*CpACO2B*, *CpACS11A*, and *CpACS27A*), ethylene perception and signaling (*CpETR1A* and *CpEIN3A*), and coding for the transcription factors (*CpWIP1B*) that are known to be involved in sex determination in cucurbit species. The relative level of each transcript was quantified by quantitative PCR in three independent replicates of each tissue. FFB, female floral bud excluding the ovary; MFB, male floral bud. Error bars represent SE. Different letters indicate significant differences between WT and mutant apical shoots and flowers at the same stage of development (*p* ≤ 0.05).

The possible regulation of *CpACO1A* over other sex-determining genes was investigated by assessing the expression of those genes in WT and *aco1a* pistillate and male flowers (Figure 7). The ethylene biosynthesis genes *CpACO2B*, *CpACS11A*, and *CpACS27A*, which are expressed at early stages of female flower development and make the floral meristem to be determined as a female flower (Martínez and Jamilena, 2021), were differentially expressed in WT and *aco1a* pistillate flowers, but not in the apical shoots or in male flowers (Figure 7). In very small floral buds (1 mm), the *aco1a* mutation repressed the expression of the three genes. In 2–3 mm floral buds, the expression of the *CpACS11A* and *CpACS27A* was repressed in the ovary, and the expression of *CpACO2B* and *CpACS11A* was induced in the rest of the floral organs (petals, style, and stigma). In 5–8 mm female floral buds, the expressions of these three ethylene biosynthesis genes were not altered by *aco1a* (Figure 7).

The mutation *aco1a* also diminished the expression of the ethylene receptor *CpETR1A* and the ethylene signaling gene *CpEIN3A* (Figure 7) at early stages of female flower development (female floral buds of 1 mm and 2–3 mm in length) and in ovaries of flowers 2–3 mm in length. In the rest of the analyzed tissues, including the apical shoot, female flowers at later stages of development and male flowers, no difference was found in the expression of these two ethylene signaling genes between WT and *aco1a* tissues (Figure 7). The expression *CpWIP1B*, a homolog of melon *WIP1* involved in the arrest of stamen during the development of male flowers, was unaltered by the mutation in most of the studied tissues, but in the apical shoot and in male floral buds of 5–8 mm in length, the gene was induced in the mutant.

### Hormone Imbalance in Early Female Development of *aco1a* Flowers

To examine whether the mutation *aco1a* can change the hormonal balance of pistillate flower, we proceeded to compare phytohormone contents of WT female flower and *aco1a* hermaphrodite flowers. Table 2 shows phytohormone concentrations of pistillate flower buds of 5–8 mm from WT and *aco1a* plants. No difference was detected for IBA, GA3, and BA contents. However, the *aco1a* flowers showed a considerable reduction in the content of ABA and JA, as well as SA (Table 2). In contrast, the auxin (IAA) content in the *aco1a* hermaphrodite flowers was much higher than that in female WT flowers (Table 2).

**TABLE 2:** Hormone concentrations ng/mL (ppb).

Hormones	WT	*aco1a*
Salicylic acid (SA)	4661.94 ± 41.00 **a**	3196.83 ± 46.34 **b**
Indole-3-butyric acid (IBA)	n.d	n.d
Indole-3-acetic acid (IAA)	<LOQ **b**	23.25 ± 2.47 **a**
Gibberellic acid (GA3)	n.d	n.d
6-Benzyladenine (BA)	n.d	n.d
Abscisic acid (ABA)	124.81 ± 4.06 **a**	36.02 ± 2.35 **b**
Jasmonic acid (JA)	656.65 ± 19.11 **a**	248.56 ± 5.41 **b**

*Different letters within the same row indicate significant differences between WT and aco1a hormone content (p < 0.05); n = 3. LOQ, results below the limit of quantification (5 ppb). n.d, not detected.*

## Discussion

It has been assumed that not ACO, but ACS, is the rate-limiting enzyme in ethylene biosynthesis. However, there is an increasing amount of evidence demonstrating the importance of ACO in controlling ethylene production in plants (Houben and Van de Poel, 2019). In cucurbits, mutations in *CmACO1* are known to inhibit fruit ripening and extend fruit shelf life (Dahmani-Mardas et al., 2010). An essential role of *CsACO2* and *CmACO3* orthologs in carpel development has been recently reported in cucumber and melon (Chen et al., 2016). In this paper, we establish that *CpACO1A* is a key regulator in sex determination and female flower development of *C. pepo*.

### *aco1a* Disrupts Ethylene Biosynthesis and Hormonal Balance During Female Flower Development

The ACO protein family can be divided in three phylogenetic groups based on amino-acid sequence similarity (Houben and Van de Poel, 2019). At a functional level, the ACO protein has two highly conserved and well-distinguished domains, one N-terminal, highly conservative non-heme dioxygenase DIOX_N region and a C-terminally located 2OG-FeII_Oxy region, both of which are critical for ACO activity (Ruduś et al., 2013). The sequence alignment and the phylogenetic tree constructed by using ACO proteins from diverse plant species have proven that CpACO1A is a type I ACO enzyme, and that the *aco1a* P5L mutation affects the first amino acid of the CpACO1A DIOX_N domain, which is a conserved proline residue in all analyzed plant ACOs. The reduced ACO activity and ethylene production in *aco1a* plant organs confirmed the disfunction of P5L isoform of CpACO1A and the importance of 5P residue for its activity.

*CpACO1A* transcript differentially accumulated in different tissues and stages of development. Comparison of ethylene production and gene expression in WT and *aco1a* organs indicated that *CpACO1A* may be regulated by ethylene in a tissue- and temporal-specific manner. This feedback regulation could also affect other ethylene biosynthesis genes involved in flower development and sex determination, including *CpACO2B*, *CpACS11A*, and *CpACS27A*. Both positive and negative feedback ethylene-mediated regulation of *ACS* and *ACO* transcription have been reported in other systems in a tissue- and temporal-specific manner during flower and fruit development (Barry et al., 1996; Nakatsuka et al., 1998; Inaba et al., 2007; Trivellini et al., 2011; Houben and Van de Poel, 2019; Pattyn et al., 2020). However, we do not exclude the possibility that the regulation of *ACS* and *ACO* genes in the female flower is mediated by other hormones, such as IAA, which was found to be highly accumulated in the ethylene-deficient *aco1a* pistillate flowers.

The hormonal imbalance detected in *aco1a* female flowers reveals the existence of crosstalk between ethylene and other hormones, such as IAA, SA, ABA, and JA, during female flower development. The coaction of ethylene and auxin has been reported in various growth and developmental processes, including root elongation, lateral root formation, hypocotyl growth, and fruit development and ripening, where both hormones may act synergistically or antagonistically (Stepanova et al., 2007; Muday et al., 2012; Li et al., 2016; Yue et al., 2020). The reciprocal positive regulation between auxin and ethylene is well established; elevated levels of auxin trigger transcriptional activation of subsets of *ACS* and *ACO* genes, leading to increased ethylene production; and ethylene positively controls IAA biosynthesis by the up-regulation of *Weak ET Insensitive 2* (*WEI2*) and *WEI7* (Růžička et al., 2007; Stepanova et al., 2007; Swarup et al., 2007; Zemlyanskaya et al., 2018). However, ethylene has also been reported to negatively regulate auxin biosynthesis (Harkey et al., 2018; Li et al., 2018). We found that ethylene and auxin are mutually repressed, likely having an antagonistic action in squash female flower development. Auxin down-regulates the expression of ethylene biosynthesis and signaling genes in the female flower upon fruit set (Martínez et al., 2013), and here we demonstrated that ethylene has a negative regulation on auxin in female flowers, accumulating much higher content of IAA in ethylene-deficient *aco1a* than in WT. On the other hand, the reduced levels of ABA, JA, and SA in the ethylene-deficient mutant *aco1a* indicates that ethylene positively regulates the homeostasis of these three phytohormones in the female flower. As discussed below, all of these hormones have key functions in flower development (Chandler, 2011), and can cooperate with ethylene in the regulation of squash female flower development.

### *CpACO1A* Prevents Stamen Development in Squash Female Flowers

Different sex-determining mechanisms prevent the development of either the stamens or the carpel in a primarily hermaphrodite floral meristem (Martínez and Jamilena, 2021). In Cucurbitaceae, ethylene arrests the development of stamens and promotes the development of carpels during the determination of female flowers. Early ethylene biosynthesis genes, such as *ACS11* and *ACO2*, in cucumber and melon are able to promote carpel development and determine the fate of floral meristem toward a female flower. The LOF mutation in these two ethylene biosynthesis genes leads to androecy in both cucumber and melon (Boualem et al., 2015; Chen et al., 2016), as occurs with mutation in some ethylene receptor genes (García et al., 2020a,b). Our results demonstrate that *aco1a* mutation led to a reduction in ACO activity and ethylene production, but induced the expression of *CpACO1A*. This upregulation also occurs for *CpACO2B* and *CpACS11A* in *aco1a*, but the induction of these two genes occurs in flowers where sex determination has already taken place (flowers above 2–3 mm in length). At earlier stages of female flower development (female floral buds less than 1 mm), the genes *CpACO2B* and *CpACS11A* were down-regulated in the mutant, and could not compensate for the reduced ethylene caused by *CpACO1A* disfunction.

The later-acting ethylene biosynthesis gene *ACS2* is specifically expressed in female flowers at early stages of development to control the arrest of stamen development. LOF mutations for *ACS2* orthologs (*CsACS2* in cucumber, *CmACS7* in melon, *CpACS27A* in *C. pepo*, and *CitACS4* in watermelon) promote the conversion of female into hermaphrodite flowers and monoecy into andromonoecy (Boualem et al., 2008, 2009, 2016; Martínez et al., 2014; Ji et al., 2016; Manzano et al., 2016). The phenotype of *aco1a* mutant described in this paper resembles those of *acs2-like* mutants, indicating that *CpACO1A* is, together with *CpACS27A*, the key enzymes that produce the requisite ethylene to prevent the development of stamens in squash female flowers. The reduced expression of *CpACS27A* in *aco1a* pistillate flowers at early stages of development suggests that the regulation of these two key enzymes is coordinated, producing the required ethylene for the proper development of the female flower. This coordinated regulation may be mediated by ethylene, as occurs in other systems (Barry et al., 2000; Inaba et al., 2007).

### *CpACO1A* Controls Flower Opening and Ovary Development in the Absence of Pollination

**FIGURE 8:**
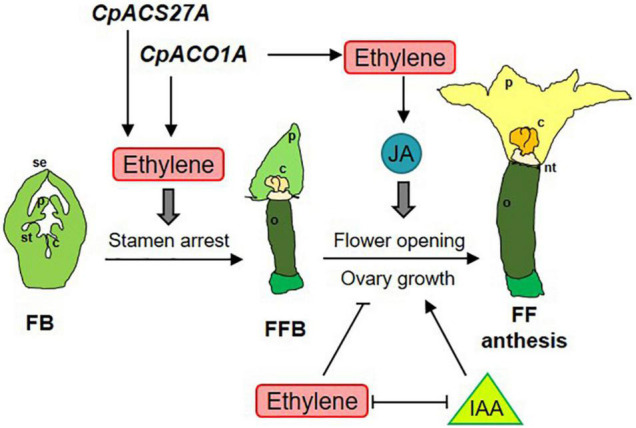
Model integrating ethylene, jasmonate (JA) and auxin (IAA) in the regulation of sex determination (stamen arrest), female flower opening and ovary growth and fruit set and development in *Cucurbita pepo*. The genes *CpACO1A* and *CpACS27A* participate in the biosynthesis of the ethylene required for carpel promotion and stamen arrest at earlier stages of female floral bud (FFB) development. *CpACO1A* also participates in the ethylene produced at later stages of FFB for controlling flower opening and ovary growth and fruit set. Petal maturation and female flower opening is mediated by jasmonic acid (JA), while ovary growth and fruit set is coordinated by the antagonist action of ethylene and IAA, which are mutually repressed during the development of the female flower. se, sepal; p, petal; st, stamen; c, carpel; o, ovary; nt, nectary; FB, floral bud, FFB, female floral bud, FF, female flower.

The phenotype of *aco1a* flowers also indicates that ethylene regulates the growth and development of other floral organs in the pistillate flowers of squash, including the corolla and the ovary/fruit. The delayed anthesis time of the *aco1a* pistillate flower demonstrates that ethylene is a positive regulator of petal growth and maturation in squash. This was also found in squash ethylene-insensitive mutants (García et al., 2020a,b), and seems to be associated with pistillate flower masculinization. Ethylene, which is the hormone that activates the developmental program of a female flower, is also used to promote the growing rate and maturation of female corolla. In the male flower, where ethylene production is very low, petals develop slower and anthesis is markedly more delayed. Given that male flowers are produced in the first nodes of the plant, this ethylene-mediated mechanism ensures that male and female flowers reach anthesis at the same time to achieve successful pollination. JA is known to be involved in anther and pollen maturation (Stintzi and Browse, 2000; Wang et al., 2005; Browse and Wallis, 2019), but also participates in petal maturation and flower opening (Reeves et al., 2012; Oh et al., 2013; Niwa et al., 2018; Schubert et al., 2019). The delayed flower opening and reduced JA in the ethylene-deficient hermaphrodite flowers of *aco1a* indicate that ethylene can regulate the maturation and opening of the female flower by inducing the biosynthesis of JA (Figure 8).

We have previously reported that external treatments with ethylene inhibitors were able induce fruit set and early fruit development in the absence of pollination (parthenocarpic fruit), and that fruit set is concomitant with a reduction in ethylene production, ethylene biosynthesis, and signaling gene expression in the days immediately after anthesis (Martínez et al., 2013). Mutations in ethylene receptor genes of squash confer partial ethylene insensitivity, and also result in parthenocarpic fruits (García et al., 2020a,b). The negative role of ethylene in fruit set has been also found in tomato, where ethylene and signaling genes are down-regulated in early-developing fruit (Vriezen et al., 2008; Wang et al., 2009), and the blocking of ethylene perception, using the ethylene-insensitive mutation *Sletr1-1* or treatments with 1MCP, leads to parthenocarpic fruits through the induction of auxin and gibberellin (Wang et al., 2009; Shinozaki et al., 2015, 2018; An et al., 2020). The up-regulation of IAA in *aco1a* may be responsible for the continued growth of *aco1a* ovaries in the absence of pollination. Auxins are the key hormones regulating fruit set in the Cucurbitaceae family (Trebitsh et al., 1987; Kim et al., 1992; Martínez et al., 2013), and were found to be highly induced in the *aco1a* flowers. This means that auxins not only repress the production, perception and signaling of ethylene in the squash developing fruit, as reported by Martínez et al. (2013), but can be negatively regulated by ethylene in the developing ovary (Figure 8). It is feasible that the two hormones are specifically accumulated in different floral organs, i.e., ethylene in the upper flower organs for promoting the development of carpels and petals and arresting the development of stamens, and auxin in the inferior ovary for inducing fruit set and development (Figure 8).

## Data Availability Statement

The datasets presented in this study can be found in online repositories. The names of the repository/repositories and accession number(s) can be found in the article/Supplementary Material.

## Author Contributions

MJ designed and coordinated the research. GC conducted most of the experiments and data analysis. JI-M and JR collaborated in phenotyping. CM and DG collaborated in data analysis. MJ and GC wrote the first version of the manuscript, and the other authors contributed later to improve it and approved the final version for submission. All authors contributed to the article and approved the submitted version.

## Conflict of Interest

The authors declare that the research was conducted in the absence of any commercial or financial relationships that could be construed as a potential conflict of interest.

## Publisher’s Note

All claims expressed in this article are solely those of the authors and do not necessarily represent those of their affiliated organizations, or those of the publisher, the editors and the reviewers. Any product that may be evaluated in this article, or claim that may be made by its manufacturer, is not guaranteed or endorsed by the publisher.

## Footnotes

1
https://international.neb.com

2
http://cucurbitgenomics.org

3
http://www.lgcgroup.com

4
https://afly.co/xyn2

5
https://blast.ncbi.nlm.nih.gov/Blast.cgi

6
https://www.rcsb.org

7
https://swissmodel.expasy.org

8
https://www.arabidopsis.org

9
http://cucurbitgenomics.org

10
https://shigen.nig.ac.jp/rice/oryzabase

11
https://solgenomics.net

12
http://gsds.gao-lab.org/Gsds_about.php

13
https://rostlab.org/services/snap2web

14
https://folding.biofold.org/i-mutant/i-mutant2.0.html

15
http://mupro.proteomics.ics.uci.edu

16
http://cupsat.tu-bs.de
