# Supplementary material for: The Ethylene Biosynthesis Gene CpACO1A: A New Player in the Regulation of Sex Determination and Female Flower Development in Cucurbita pepo
Source: Front Plant Sci. 2022 Jan 24;12:817922. doi: 10.3389/fpls.2021.817922 (PMC8818733; doi:10.3389/fpls.2021.817922)
Supplement: Supplementary file 1 [file Table_1.DOCX]

| **Table S1.** List of the *ACO* genes and proteins used in phylogenetic analysis. | | | | |
| --- | --- | --- | --- | --- |
| **Species** | **GeneID** | **Gene** | **Type** | **Protein (aa)** |
| ***Cucurbita pepo*** | Cp4.1LG04g02610  Cp4.1LG05g15190  Cp4.1LG10g09730  Cp4.1LG00g09300  Cp4.1LG19g08030  Cp4.1LG02g03090  Cp4.1LG06g06110  Cp4.1LG07g10650 | *CpACO1A*  *CpACO1B*  *CpACO2A-1*  *CpACO2A-2*  *CpACO2B*  *CpACO5A*  *CpACO5B*  *CpACO5-Like* | 1  1  1  1  1  3  3  2 | 319  311  316  334  336  311  309  299 |
| ***Cucumis melo*** | MELO3C014437  MELO3C007425  MELO3C019735  MELO3C010508  MELO3C004619 | *CmACO1*  *CmACO3*  *-*  *-*  *-* | 1  1  1  3  2 | 318  327  314  309  300 |
| ***Cucumis sativus*** | Csa6G160180  Csa6G511860  Csa6G421630  Csa4G361270  Csa2G000520 | *CsACO1*  *CsACO2*  *CsACO3*  *CsACO4*  *CsACO5* | 1  1  1  3  2 | 314  320  317  309  300 |
| ***Arabidopsis thaliana*** | AT2G19590.1  AT1G62380.1  AT1G12010.1  AT1G05010.1  AT1G77330.1 | *AtACO1*  *AtACO2*  *AtACO3*  *AtACO4*  *AtACO5* | 2  1  1  1  3 | 308  320  320  323  307 |
| ***Solanum lycopersicum*** | Solyc07g049530.2.1  Solyc12g005940.1.1  Solyc07g049550.2.1  Solyc02g081190.2.1  Solyc07g026650.2.1  Solyc02g036350.2.1  Solyc06g060070.2.1 | *SlACO1*  *SlACO2*  *SlACO3*  *SlACO4*  *SlACO5*  *SlACO6*  *SlACO7* | 1  1  1  1  2  1  3 | 315  316  316  320  301  319  314 |
| ***Oryza sativa*** | LOC_Os09g27750.1  LOC_Os02g53180.1  LOC_Os02g53180.2  LOC_Os02g53180.3  LOC_Os06g37590.1  LOC_Os01g39860.1  LOC_Os11g08380.1  LOC_Os05g05680.1 | *OsACO2*  *OsACO3α*  *OsACO3β*  *OsACO3γ*  *OsACO6*  *OsACO7*  *OsACO4*  *OsACO5* | 1  1  1  1  2  2  3  3 | 323  345  322  284  294  313  310  309 |

| **Table S2.** Primers used for gene expression analysis by qPCR. | | |
| --- | --- | --- |
| **Gene** | **Primer** | **Sequence** |
| ***CpACO1A*** | F | CATAGAGTGATGACTCAGACAAGC |
|  | R | CCATTGGACCCAAATTAGCA |
| ***CpACO1B*** | F | CAGTTGCTGAAAGACGACCA |
|  | R | TGGTCATATATTCCCCTGAGTTG |
| ***CpACS11A*** | F | CGTCGTCTTAAGGCCTTTG |
|  | R | GGTGTACCTAATTTAACGCAAC |
| ***CpACS27A*** | F | CCAATACGGACGGTGAA |
|  | R | GGAGAAGCTGAAGAAGGAAG |
| ***CpACO2B*** | F | GGAGGGAGAGGAAGATAAGG |
|  | R | TGGGTTATTGGAAAATGGAG |
| ***CpWIP1B*** | F | TCCCTACTCCATGCTTCAC |
|  | R | TCCTCCTCATTCACAACAAC |
| ***CpETR1A*** | F | AAAGGAGAGCTGCCTGAGAGTC |
|  | R | CACGACGCTCTATAAGTTCCGA |
| ***CpEIN3A*** | F | TAGCAGCCAATTCAACCAGTTTAAGCC |
|  | R | CGGTAAAGCATCGAATTGAGATCAGG |
| ***CpEf-1α*** | F | CGTCAAGAAGAAATAAGCCA |
|  | R | CTACTACGAGAGAGAGAGCCG |

| **Table S3**. Inheritance of *aco1a* mutant in the backcrossing and selfing generations. | | | | | |
| --- | --- | --- | --- | --- | --- |
|  | **Number of plants** | |  | |  |
| **Generation** | **WT** | ***aco1a*** | **Expected segregation** | **Χ_c_²** | **p-value** |
| BC_1_ | 43 | - | 1:0 | - | - |
| BC_1_S_1_ | 163 | 57 | 3:1 | 0.10 | > 0.05 |
| BC_2_S_1_ | 219 | 81 | 3:1 | 0.64 | > 0.05 |

| **Table S4.** Validation of the identified mutations by high-throughput genotyping of individual segregating plants. | | | | | | | | | | | | |
| --- | --- | --- | --- | --- | --- | --- | --- | --- | --- | --- | --- | --- |
| **Plant** | **Phenotype** | **Genotype** | **Andromonoecius Index (AI)** | | | | | | | | | |
| 1 | WT | wt/wt | 0 | 0 | 0 | 0 | 0 | 0 | 0 | 0 | 0 | 0 |
| 2 | WT | wt/*aco1a* | 0 | 0 | 0 | 0 | 0 | 0 | 0 | 0 | 0 | 0 |
| 3 | *aco1a* | *aco1a/aco1a* | **1,5** | **0,5** | **0,5** | **0,5** | **2,5** | **2,8** | **2** | **1,8** | **2,8** | **3** |
| 4 | WT | wt/wt | 0 | 0 | 0 | 0 | 0 | 0 | 0 | 0 | 0 | 0 |
| 5 | WT | wt/*aco1a* | 0 | 0 | 0 | 0 | 0 | 0 | 0 | 0 | 0 | 0 |
| 6 | WT | wt/*aco1a* | 0 | 0 | 0 | 0 | 0 | 0 | 0 | 0 | 0 | 0 |
| 7 | WT | wt/wt | 0 | 0 | 0 | 0 | 0 | 0 | 0 | 0 | 0 | 0 |
| 8 | *aco1a* | *aco1a/aco1a* | **3** | **3** | **0,5** | **1** | **1** | **2** | **1** | **3** | **3** | **3** |
| 9 | WT | wt/*aco1a* | 0 | 0 | 0 | 0 | 0 | 0 | 0 | 0 | 0 | 0 |
| 10 | WT | wt/*aco1a* | 0 | 0 | 0 | 0 | 0 | 0 | 0 | 0 | 0 | 0 |
| 11 | WT | wt/wt | 0 | 0 | 0 | 0 | 0 | 0 | 0 | 0 | 0 | 0 |
| 12 | WT | wt/wt | 0 | 0 | 0 | 0 | 0 | 0 | 0 | 0 | 0 | 0 |
| 13 | WT | wt/*aco1a* | 0 | 0 | 0 | 0 | 0 | 0 | 0 | 0 | 0 | 0 |
| 14 | WT | wt/*aco1a* | 0 | 0 | 0 | 0 | 0 | 0 | 0 | 0 | 0 | 0 |
| 15 | WT | wt/wt | 0 | 0 | 0 | 0 | 0 | 0 | 0 | 0 | 0 | 0 |
| 16 | *aco1a* | *aco1a/aco1a* | **3** | **3** | **0,5** | **0,5** | **1** | **1,5** | **2,5** | **2** | **1,5** | **2,5** |
| 17 | WT | wt/*aco1a* | 0 | 0 | 0 | 0 | 0 | 0 | 0 | 0 | 0 | 0 |
| 18 | WT | wt/*aco1a* | 0 | 0 | 0 | 0 | 0 | 0 | 0 | 0 | 0 | 0 |
| 19 | WT | wt/*aco1a* | 0 | 0 | 0 | 0 | 0 | 0 | 0 | 0 | 0 | 0 |
| 20 | WT | wt/wt | 0 | 0 | 0 | 0 | 0 | 0 | 0 | 0 | 0 | 0 |
| 21 | WT | wt/wt | 0 | 0 | 0 | 0 | 0 | 0 | 0 | 0 | 0 | 0 |
| 22 | *aco1a* | *aco1a/aco1a* | **3** | **3** | **1** | **1,5** | **0,5** | **2,8** | **3** | **3** | **3** | **3** |
| 23 | WT | wt/*aco1a* | 0 | 0 | 0 | 0 | 0 | 0 | 0 | 0 | 0 | 0 |
| 24 | WT | wt/*aco1a* | 0 | 0 | 0 | 0 | 0 | 0 | 0 | 0 | 0 | 0 |
| 25 | WT | wt/wt | 0 | 0 | 0 | 0 | 0 | 0 | 0 | 0 | 0 | 0 |
| 26 | WT | wt/*aco1a* | 0 | 0 | 0 | 0 | 0 | 0 | 0 | 0 | 0 | 0 |
| 27 | WT | wt/wt | 0 | 0 | 0 | 0 | 0 | 0 | 0 | 0 | 0 | 0 |
| 28 | *aco1a* | *aco1a/aco1a* | **2** | **2** | **2,5** | **1** | **2,5** | **2** | **2,5** | **3** | **3** | **3** |
| 29 | *aco1a* | *aco1a/aco1a* | **0,5** | **1** | **1** | **1** | **1** | **2** | **3** | **1,8** | **2,5** | **2,3** |
| 30 | WT | wt/*aco1a* | 0 | 0 | 0 | 0 | 0 | 0 | 0 | 0 | 0 | 0 |
| 31 | WT | wt/wt | 0 | 0 | 0 | 0 | 0 | 0 | 0 | 0 | 0 | 0 |
| 32 | WT | wt/*aco1a* | 0 | 0 | 0 | 0 | 0 | 0 | 0 | 0 | 0 | 0 |
| 33 | *aco1a* | *aco1a/aco1a* | **0** | **1** | **1,5** | **2** | **1,5** | **1** | **2,5** | **1** | **2** | **3** |
| 34 | WT | wt/*aco1a* | 0 | 0 | 0 | 0 | 0 | 0 | 0 | 0 | 0 | 0 |
| 35 | WT | wt/*aco1a* | 0 | 0 | 0 | 0 | 0 | 0 | 0 | 0 | 0 | 0 |
| 36 | WT | wt/wt | 0 | 0 | 0 | 0 | 0 | 0 | 0 | 0 | 0 | 0 |
| 37 | *aco1a* | *aco1a/aco1a* | **1,5** | **1** | **1** | **1,5** | **2,5** | **1** | **3** | **3** | **3** | **3** |
| 38 | WT | wt/*aco1a* | 0 | 0 | 0 | 0 | 0 | 0 | 0 | 0 | 0 | 0 |
| 39 | *aco1a* | *aco1a/aco1a* | **0** | **1,5** | **0,5** | **1** | **1,5** | **0,5** | **1** | **2** | **2,5** | **2** |
| 40 | WT | wt/*aco1a* | 0 | 0 | 0 | 0 | 0 | 0 | 0 | 0 | 0 | 0 |
| 41 | WT | wt/wt | 0 | 0 | 0 | 0 | 0 | 0 | 0 | 0 | 0 | 0 |
| 42 | WT | wt/wt | 0 | 0 | 0 | 0 | 0 | 0 | 0 | 0 | 0 | 0 |
| 43 | WT | wt/*aco1a* | 0 | 0 | 0 | 0 | 0 | 0 | 0 | 0 | 0 | 0 |
| 44 | WT | wt/*aco1a* | 0 | 0 | 0 | 0 | 0 | 0 | 0 | 0 | 0 | 0 |
| 45 | WT | wt/*aco1a* | 0 | 0 | 0 | 0 | 0 | 0 | 0 | 0 | 0 | 0 |
| 46 | *aco1a* | *aco1a/aco1a* | **3** | **2** | **1** | **1** | **1** | **1,5** | **2** | **3** | **2,8** | **3** |
| 47 | WT | wt/*aco1a* | 0 | 0 | 0 | 0 | 0 | 0 | 0 | 0 | 0 | 0 |
| 48 | WT | wt/*aco1a* | 0 | 0 | 0 | 0 | 0 | 0 | 0 | 0 | 0 | 0 |
| 49 | *aco1a* | *aco1a/aco1a* | **0,5** | **0,5** | **2** | **0,5** | **0,5** | **1** | **1,5** | **1** | **0,5** | **1,5** |
| 50 | *aco1a* | *aco1a/aco1a* | **0,5** | **1** | **2** | **1** | **0,5** | **0,5** | **1** | **2,5** | **0,5** | **0,5** |
| 51 | WT | wt/*aco1a* | 0 | 0 | 0 | 0 | 0 | 0 | 0 | 0 | 0 | 0 |
| 52 | *aco1a* | *aco1a/aco1a* | **2,5** | **1,5** | **2** | **0,5** | **0,5** | **2,5** | **0,5** | **3** | **3** | **3** |
| 53 | WT | wt/*aco1a* | 0 | 0 | 0 | 0 | 0 | 0 | 0 | 0 | 0 | 0 |
| 54 | WT | wt/*aco1a* | 0 | 0 | 0 | 0 | 0 | 0 | 0 | 0 | 0 | 0 |
| 55 | *aco1a* | *aco1a/aco1a* | **2,5** | **2** | **1** | **0,5** | **1** | **1** | **1** | **1,5** | **1,5** | **2** |
| 56 | WT | wt/*aco1a* | 0 | 0 | 0 | 0 | 0 | 0 | 0 | 0 | 0 | 0 |
| 57 | *aco1a* | *aco1a/aco1a* | **3** | **1,5** | **1** | **1** | **0,5** | **0,5** | **0,5** | **3** | **3** | **3** |
| 58 | WT | wt/wt | 0 | 0 | 0 | 0 | 0 | 0 | 0 | 0 | 0 | 0 |
| 59 | WT | wt/*aco1a* | 0 | 0 | 0 | 0 | 0 | 0 | 0 | 0 | 0 | 0 |
| 60 | WT | wt/wt | 0 | 0 | 0 | 0 | 0 | 0 | 0 | 0 | 0 | 0 |
| 61 | *aco1a* | *aco1a/aco1a* | **2,5** | **0,5** | **0,5** | **0,5** | **0,5** | **2** | **1,5** | **2,5** | **1,5** | **2,8** |
| 62 | *aco1a* | *aco1a/aco1a* | **0** | **0,5** | **1** | **0,5** | **1** | **1** | **0,5** | **0,5** | **1** | **2,8** |
| 63 | *aco1a* | *aco1a/aco1a* | **0,5** | **3** | **2,5** | **1,5** | **1** | **3** | **3** | **3** | **2,8** | **3** |
| 64 | WT | wt/wt | 0 | 0 | 0 | 0 | 0 | 0 | 0 | 0 | 0 | 0 |
| 65 | WT | wt/*aco1a* | 0 | 0 | 0 | 0 | 0 | 0 | 0 | 0 | 0 | 0 |
| 66 | WT | wt/*aco1a* | 0 | 0 | 0 | 0 | 0 | 0 | 0 | 0 | 0 | 0 |
| 67 | *aco1a* | *aco1a/aco1a* | **2** | **1** | **0,5** | **0,5** | **0,5** | **3** | **2,8** | **3** | **3** | **2,8** |
| 68 | *aco1a* | *aco1a/aco1a* | **2,5** | **1** | **1** | **0,5** | **1,5** | **3** | **2,5** | **2,8** | **3** | **2,5** |
| 69 | *aco1a* | *aco1a/aco1a* | **1** | **1,5** | **1,5** | **1** | **1** | **0,5** | **1,5** | **1** | **2** | **2,5** |
| 70 | WT | wt/*aco1a* | 0 | 0 | 0 | 0 | 0 | 0 | 0 | 0 | 0 | 0 |
| 71 | *aco1a* | *aco1a/aco1a* | **0,5** | **2** | **0,5** | **1** | **0,5** | **0,5** | **0** | **1** | **0,5** | **2,5** |
| 72 | WT | wt/*aco1a* | 0 | 0 | 0 | 0 | 0 | 0 | 0 | 0 | 0 | 0 |
| 73 | WT | wt/*aco1a* | 0 | 0 | 0 | 0 | 0 | 0 | 0 | 0 | 0 | 0 |
| 74 | WT | wt/*aco1a* | 0 | 0 | 0 | 0 | 0 | 0 | 0 | 0 | 0 | 0 |
| 75 | WT | wt/wt | 0 | 0 | 0 | 0 | 0 | 0 | 0 | 0 | 0 | 0 |
| 76 | *aco1a* | *aco1a/aco1a* | **3** | **3** | **2,5** | **2,5** | **2** | **1,8** | **2,8** | **2,5** | **2,8** | **3** |
| 77 | WT | wt/wt | 0 | 0 | 0 | 0 | 0 | 0 | 0 | 0 | 0 | 0 |
| 78 | WT | wt/wt | 0 | 0 | 0 | 0 | 0 | 0 | 0 | 0 | 0 | 0 |
| 79 | WT | wt/*aco1a* | 0 | 0 | 0 | 0 | 0 | 0 | 0 | 0 | 0 | 0 |
| 80 | *aco1a* | *aco1a/aco1a* | **3** | **1** | **1** | **2,5** | **1,8** | **1,8** | **2** | **1,8** | **1,8** | **2,8** |
| 81 | WT | wt/*aco1a* | 0 | 0 | 0 | 0 | 0 | 0 | 0 | 0 | 0 | 0 |
| 82 | *aco1a* | *aco1a/aco1a* | **1,5** | **2** | **1** | **0,5** | **1** | **1** | **1,5** | **1,8** | **2,5** | **3** |
| 83 | WT | wt/wt | 0 | 0 | 0 | 0 | 0 | 0 | 0 | 0 | 0 | 0 |
| 84 | WT | wt/*aco1a* | 0 | 0 | 0 | 0 | 0 | 0 | 0 | 0 | 0 | 0 |
| 85 | *aco1a* | *aco1a/aco1a* | **1,5** | **1,5** | **2** | **2** | **2** | **3** | **3** | **3** | **3** | **2** |
| 86 | WT | wt/*aco1a* | 0 | 0 | 0 | 0 | 0 | 0 | 0 | 0 | 0 | 0 |
| 87 | *aco1a* | *aco1a/aco1a* | **2,5** | **0,5** | **1** | **1,5** | **1,5** | **0,5** | **3** | **2,8** | **1,5** | **3** |
| 88 | WT | wt/*aco1a* | 0 | 0 | 0 | 0 | 0 | 0 | 0 | 0 | 0 | 0 |
| 89 | WT | wt/wt | 0 | 0 | 0 | 0 | 0 | 0 | 0 | 0 | 0 | 0 |
| 90 | WT | wt/*aco1a* | 0 | 0 | 0 | 0 | 0 | 0 | 0 | 0 | 0 | 0 |
| 91 | WT | wt/wt | 0 | 0 | 0 | 0 | 0 | 0 | 0 | 0 | 0 | 0 |
| 92 | WT | wt/*aco1a* | 0 | 0 | 0 | 0 | 0 | 0 | 0 | 0 | 0 | 0 |
| 93 | WT | wt/*aco1a* | 0 | 0 | 0 | 0 | 0 | 0 | 0 | 0 | 0 | 0 |
| 94 | WT | wt/wt | 0 | 0 | 0 | 0 | 0 | 0 | 0 | 0 | 0 | 0 |
| 95 | *aco1a* | *aco1a/aco1a* | **1** | **1,5** | **1** | **2** | **1,5** | **3** | **2** | **1,5** | **1** | **3** |
| 96 | *aco1a* | *aco1a/aco1a* | **3** | **3** | **1** | **1** | **1,5** | **1** | **1** | **3** | **2,5** | **3** |
| 97 | WT | wt/*aco1a* | 0 | 0 | 0 | 0 | 0 | 0 | 0 | 0 | 0 | 0 |
| 98 | WT | wt/wt | 0 | 0 | 0 | 0 | 0 | 0 | 0 | 0 | 0 | 0 |
| 99 | WT | wt/wt | 0 | 0 | 0 | 0 | 0 | 0 | 0 | 0 | 0 | 0 |
| 100 | *aco1a* | *aco1a/aco1a* | **0,5** | **1** | **3** | **0,5** | **1,5** | **0,5** | **0,5** | **3** | **2,8** | **3** |
| 101 | WT | wt/*aco1a* | 0 | 0 | 0 | 0 | 0 | 0 | 0 | 0 | 0 | 0 |
| 102 | WT | wt/wt | 0 | 0 | 0 | 0 | 0 | 0 | 0 | 0 | 0 | 0 |
| 103 | WT | wt/*aco1a* | 0 | 0 | 0 | 0 | 0 | 0 | 0 | 0 | 0 | 0 |
| 104 | *aco1a* | *aco1a/aco1a* | **3** | **0,5** | **0,5** | **2,8** | **3** | **3** | **3** | **3** | **3** | **3** |
| 105 | WT | wt/*aco1a* | 0 | 0 | 0 | 0 | 0 | 0 | 0 | 0 | 0 | 0 |
| 106 | WT | wt/*aco1a* | 0 | 0 | 0 | 0 | 0 | 0 | 0 | 0 | 0 | 0 |
| 107 | *aco1a* | *aco1a/aco1a* | **0** | **1** | **0,5** | **0,8** | **0,8** | **3** | **3** | **1,8** | **3** | **3** |
| 108 | *aco1a* | *aco1a/aco1a* | **3** | **1,5** | **1** | **1,5** | **1,5** | **2,8** | **2,8** | **3** | **1,5** | **2** |
| 109 | WT | wt/*aco1a* | 0 | 0 | 0 | 0 | 0 | 0 | 0 | 0 | 0 | 0 |
| 110 | *aco1a* | *aco1a/aco1a* | **1** | **1** | **1,5** | **1,5** | **2,8** | Determinate | | | | |
| 111 | WT | wt/*aco1a* | 0 | 0 | 0 | 0 | 0 | 0 | 0 | 0 | 0 | 0 |
| 112 | WT | wt/wt | 0 | 0 | 0 | 0 | 0 | 0 | 0 | 0 | 0 | 0 |
| 113 | WT | wt/wt | 0 | 0 | 0 | 0 | 0 | 0 | 0 | 0 | 0 | 0 |
| 114 | WT | wt/*aco1a* | 0 | 0 | 0 | 0 | 0 | 0 | 0 | 0 | 0 | 0 |
| 115 | *aco1a* | *aco1a/aco1a* | **2,5** | **1** | **1** | **0,5** | **0,5** | **0,5** | **3** | **3** | **2,8** | **3** |
| 116 | WT | wt/*aco1a* | 0 | 0 | 0 | 0 | 0 | 0 | 0 | 0 | 0 | 0 |
| 117 | WT | wt/*aco1a* | 0 | 0 | 0 | 0 | 0 | 0 | 0 | 0 | 0 | 0 |
| 118 | *aco1a* | *aco1a/aco1a* | **1,5** | **2** | **1** | **1** | **1** | **0,5** | **1** | **1,8** | **3** | **2,8** |
| 119 | WT | wt/*aco1a* | 0 | 0 | 0 | 0 | 0 | 0 | 0 | 0 | 0 | 0 |
| 120 | *aco1a* | *aco1a/aco1a* | **3** | **2** | **1** | **2,5** | **2** | **3** | **3** | **3** | **3** | **3** |
| 121 | WT | wt/wt | 0 | 0 | 0 | 0 | 0 | 0 | 0 | 0 | 0 | 0 |
| 122 | WT | wt/*aco1a* | 0 | 0 | 0 | 0 | 0 | 0 | 0 | 0 | 0 | 0 |
| 123 | WT | wt/wt | 0 | 0 | 0 | 0 | 0 | 0 | 0 | 0 | 0 | 0 |
| 124 | WT | wt/*aco1a* | 0 | 0 | 0 | 0 | 0 | 0 | 0 | 0 | 0 | 0 |
| 125 | WT | wt/wt | 0 | 0 | 0 | 0 | 0 | 0 | 0 | 0 | 0 | 0 |
| 126 | *aco1a* | *aco1a/aco1a* | **3** | **2,5** | **3** | **3** | **2,5** | **2,5** | **3** | **3** | **3** | **3** |
| 127 | WT | wt/*aco1a* | 0 | 0 | 0 | 0 | 0 | 0 | 0 | 0 | 0 | 0 |
| 128 | WT | wt/*aco1a* | 0 | 0 | 0 | 0 | 0 | 0 | 0 | 0 | 0 | 0 |
| 129 | WT | wt/*aco1a* | 0 | 0 | 0 | 0 | 0 | 0 | 0 | 0 | 0 | 0 |
| 130 | *aco1a* | *aco1a/aco1a* | **3** | **3** | **3** | **3** | **1** | **3** | **3** | **3** | **3** | **3** |
| 131 | *aco1a* | *aco1a/aco1a* | **2** | **2,5** | **2,5** | **1,5** | **1** | **2,5** | **2,8** | **3** | **0,5** | **2,5** |
| 132 | WT | wt/*aco1a* | 0 | 0 | 0 | 0 | 0 | 0 | 0 | 0 | 0 | 0 |
| 133 | WT | wt/wt | 0 | 0 | 0 | 0 | 0 | 0 | 0 | 0 | 0 | 0 |
| 134 | WT | wt/*aco1a* | 0 | 0 | 0 | 0 | 0 | 0 | 0 | 0 | 0 | 0 |
| 135 | *aco1a* | *aco1a/aco1a* | **3** | **3** | **2** | **3** | **3** | **2,8** | **3** | **3** | **3** | **3** |
| 136 | WT | wt/*aco1a* | 0 | 0 | 0 | 0 | 0 | 0 | 0 | 0 | 0 | 0 |
| 137 | WT | wt/wt | 0 | 0 | 0 | 0 | 0 | 0 | 0 | 0 | 0 | 0 |
| 138 | WT | wt/*aco1a* | 0 | 0 | 0 | 0 | 0 | 0 | 0 | 0 | 0 | 0 |
| 139 | WT | wt/*aco1a* | 0 | 0 | 0 | 0 | 0 | 0 | 0 | 0 | 0 | 0 |
| 140 | WT | wt/wt | 0 | 0 | 0 | 0 | 0 | 0 | 0 | 0 | 0 | 0 |
| 141 | WT | wt/wt | 0 | 0 | 0 | 0 | 0 | 0 | 0 | 0 | 0 | 0 |
| 142 | WT | wt/*aco1a* | 0 | 0 | 0 | 0 | 0 | 0 | 0 | 0 | 0 | 0 |
| 143 | WT | wt/*aco1a* | 0 | 0 | 0 | 0 | 0 | 0 | 0 | 0 | 0 | 0 |
| 144 | *aco1a* | *aco1a/aco1a* | **3** | **1** | **3** | **2** | **2,8** | **3** | **3** | **3** | **3** | **3** |
| 145 | *aco1a* | *aco1a/aco1a* | **2** | **1,5** | **1,5** | **3** | **3** | **3** | **3** | **1,5** | **2,8** | **3** |
| 146 | WT | wt/*aco1a* | 0 | 0 | 0 | 0 | 0 | 0 | 0 | 0 | 0 | 0 |
| 147 | WT | wt/*aco1a* | 0 | 0 | 0 | 0 | 0 | 0 | 0 | 0 | 0 | 0 |
| 148 | WT | wt/wt | 0 | 0 | 0 | 0 | 0 | Determinate | | | | |
| 149 | WT | wt/*aco1a* | 0 | 0 | 0 | 0 | 0 | 0 | 0 | 0 | 0 | 0 |
| 150 | *aco1a* | *aco1a/aco1a* | **1** | **3** | **2,5** | **3** | **3** | **2,5** | **3** | **3** | **3** | **3** |
| 151 | WT | wt/*aco1a* | 0 | 0 | 0 | 0 | 0 | 0 | 0 | 0 | 0 | 0 |
| 152 | WT | wt/*aco1a* | 0 | 0 | 0 | 0 | 0 | 0 | 0 | 0 | 0 | 0 |
| 153 | WT | wt/wt | 0 | 0 | 0 | 0 | 0 | 0 | 0 | 0 | 0 | 0 |
| 154 | WT | wt/*aco1a* | 0 | 0 | 0 | Determinate | | | | | | |
| 155 | WT | wt/*aco1a* | 0 | 0 | 0 | 0 | 0 | 0 | 0 | 0 | 0 | 0 |
| 156 | *aco1a* | *aco1a/aco1a* | **0** | **1** | **2,5** | **2** | **2,5** | **3** | **3** | **3** | **2,8** | **3** |
| 157 | WT | wt/*aco1a* | 0 | 0 | 0 | 0 | 0 | 0 | 0 | 0 | 0 | 0 |
| 158 | WT | wt/*aco1a* | 0 | 0 | 0 | 0 | 0 | 0 | 0 | 0 | 0 | 0 |
| 159 | *aco1a* | *aco1a/aco1a* | **0,5** | **2** | **1** | **0,5** | **2** | **2** | **0,8** | **3** | **3** | **3** |
| 160 | WT | wt/*aco1a* | 0 | 0 | 0 | 0 | 0 | 0 | 0 | 0 | 0 | 0 |
| 161 | *aco1a* | *aco1a/aco1a* | **2** | **2** | **1** | **0,5** | **1** | **3** | **3** | **3** | **2,8** | **2,8** |
| 162 | WT | wt/*aco1a* | 0 | 0 | 0 | 0 | 0 | 0 | 0 | 0 | 0 | 0 |
| 163 | WT | wt/*aco1a* | 0 | 0 | 0 | 0 | 0 | 0 | 0 | 0 | 0 | 0 |
| 164 | *aco1a* | *aco1a/aco1a* | **3** | **3** | **1** | **1,5** | **3** | **3** | **3** | **2,8** | **3** | **3** |
| 165 | *aco1a* | *aco1a/aco1a* | **2,5** | **1,5** | **2** | **1,5** | **1,5** | **3** | **3** | **3** | **3** | **3** |
| 166 | *aco1a* | *aco1a/aco1a* | **2,5** | **1** | **1** | **1,5** | **1,5** | **2,8** | **3** | **3** | **3** | **2,5** |
| 167 | WT | wt/wt | 0 | 0 | 0 | 0 | 0 | 0 | 0 | 0 | 0 | 0 |
| 168 | *aco1a* | *aco1a/aco1a* | **3** | **3** | **3** | **3** | **3** | **3** | **3** | **3** | **2,8** | **3** |
| 169 | WT | wt/*aco1a* | 0 | 0 | 0 | 0 | 0 | 0 | 0 | 0 | 0 | 0 |
| 170 | *aco1a* | *aco1a/aco1a* | **1** | **1,5** | **0,5** | **3** | **3** | **2** | **3** | **2,8** | **3** | **3** |
| 171 | *aco1a* | *aco1a/aco1a* | **2** | **0,5** | **0** | **2,8** | **1,8** | **1,5** | **1,5** | **2,8** | **3** | **3** |
| 172 | WT | wt/wt | 0 | 0 | 0 | 0 | 0 | 0 | 0 | 0 | 0 | 0 |
| 173 | WT | wt/*aco1a* | 0 | 0 | 0 | 0 | 0 | 0 | 0 | 0 | 0 | 0 |
| 174 | *aco1a* | *aco1a/aco1a* | **2,5** | **3** | **0,5** | **3** | **3** | **2** | **3** | **3** | **3** | **2,8** |
| 175 | WT | wt/*aco1a* | 0 | 0 | 0 | 0 | 0 | 0 | 0 | 0 | 0 | 0 |
| 176 | WT | wt/*aco1a* | 0 | 0 | 0 | 0 | 0 | 0 | 0 | 0 | 0 | 0 |
| 177 | *aco1a* | *aco1a/aco1a* | **1,5** | **1** | **1** | **2** | **3** | **3** | **2** | **2,5** | **2,5** | **3** |
| 178 | WT | wt/wt | 0 | 0 | 0 | 0 | 0 | 0 | 0 | 0 | 0 | 0 |
| 179 | *aco1a* | *aco1a/aco1a* | **3** | **3** | **2** | **3** | **3** | **3** | **2,5** | **3** | **1,5** | **2,8** |
| 180 | WT | wt/*aco1a* | 0 | 0 | 0 | 0 | 0 | 0 | 0 | 0 | 0 | 0 |
| 181 | WT | wt/*aco1a* | 0 | 0,5 | 0 | 0 | 0 | 0 | 0 | 0 | 0 | 0 |
| 182 | WT | wt/*aco1a* | 0 | 0 | 0 | 0 | 0 | 0 | 0 | 0 | 0 | 0 |
| 183 | *aco1a* | *aco1a/aco1a* | **2,5** | **0,5** | **2** | **1** | **2,5** | **2,5** | **3** | **3** | **3** | **3** |
| 184 | WT | wt/*aco1a* | 0 | 0 | 0 | 0 | 0 | 0 | 0 | 0 | 0 | 0 |
| 185 | WT | wt/*aco1a* | 0 | 0 | 0 | 0 | 0 | 0 | 0 | 0 | 0 | 0 |
| 186 | WT | wt/wt | 0 | 0 | 0 | 0 | 0 | 0 | 0 | 0 | 0 | 0 |
| 187 | WT | wt/*aco1a* | 0 | 0 | 0 | 0 | 0 | 0 | 0 | 0 | 0 | 0 |
| 188 | WT | wt/*aco1a* | 0 | 0 | 0 | 0 | 0 | 0 | 0 | 0 | 0 | 0 |
| 189 | WT | wt/wt | 0 | 0 | 0 | 0 | 0 | 0 | 0 | 0 | 0 | 0 |
| 190 | WT | wt/wt | 0 | 0 | 0 | 0 | 0 | 0 | 0 | 0 | 0 | 0 |
| 191 | WT | wt/wt | 0 | 0 | 0 | 0 | 0 | 0 | 0 | 0 | 0 | 0 |
| 192 | WT | wt/wt | 0 | 0 | 0 | 0 | 0 | 0 | 0 | 0 | 0 | 0 |
| 193 | WT | wt/wt | 0 | 0 | 0 | 0 | 0 | 0 | 0 | 0 | 0 | 0 |
| 194 | WT | wt/wt | 0 | 0 | 0 | 0 | 0 | 0 | 0 | 0 | 0 | 0 |
| 195 | WT | wt/*aco1a* | 0 | 0 | 0 | 0 | 0 | 0 | 0 | 0 | 0 | 0 |
| 196 | *aco1a* | *aco1a/aco1a* | **3** | **0,5** | **0,5** | **1** | **2** | **2,8** | **1** | **2,8** | **3** | **2,8** |
| 197 | *aco1a* | *aco1a/aco1a* | **0,5** | **1** | **0,5** | **3** | **0,5** | **2** | **2,5** | **3** | **2,8** | **3** |
| 198 | WT | wt/*aco1a* | 0 | 0 | 0 | 0 | 0 | 0 | 0 | 0 | 0 | 0 |
| 199 | WT | wt/*aco1a* | 0 | 0 | 0 | 0 | 0 | 0 | 0 | 0 | 0 | 0 |
| 200 | WT | wt/wt | 0 | 0 | 0 | 0 | 0 | 0 | 0 | 0 | 0 | 0 |
| 201 | WT | wt/wt | 0 | 0 | 0 | 0 | 0 | 0 | 0 | 0 | 0 | 0 |
| 202 | *aco1a* | *aco1a/aco1a* | **2** | **3** | **2,5** | **0,5** | **1** | **2,8** | **3** | **2,8** | **3** | **2,8** |
| 203 | WT | wt/*aco1a* | 0 | 0 | 0 | 0 | 0 | 0 | 0 | 0 | 0 | 0 |
| 204 | *aco1a* | *aco1a/aco1a* | **3** | **2,5** | **2** | **2,5** | **3** | **2,8** | **3** | **2,8** | **3** | **2** |
| 205 | WT | wt/wt | 0 | 0 | 0 | 0 | 0 | 0 | 0 | 0 | 0 | 0 |
| 206 | WT | wt/*aco1a* | 0 | 0 | 0 | 0 | 0 | 0 | 0 | 0 | 0 | 0 |
| 207 | WT | wt/*aco1a* | 0 | 0 | 0 | 0 | 0 | 0 | 0 | 0 | 0 | 0 |
| 208 | *aco1a* | *aco1a/aco1a* | **3** | **0,5** | **1** | **1** | **2,5** | **3** | **0,5** | **2,5** | **2,8** | **3** |
| 209 | *aco1a* | *aco1a/aco1a* | **3** | **0,5** | **0,5** | **0,5** | **0,5** | **3** | **1,8** | **1,5** | **1,8** | **1,8** |
| 210 | WT | wt/*aco1a* | 0 | 0 | 0 | 0 | 0 | 0 | 0 | 0 | 0 | 0 |
| 211 | WT | wt/wt | 0 | 0 | 0 | 0 | 0 | 0 | 0 | 0 | 0 | 0 |
| 212 | WT | wt/*aco1a* | 0 | 0 | 0 | 0 | 0 | 0 | 0 | 0 | 0 | 0 |
| 213 | WT | wt/wt | 0 | 0 | 0 | 0 | 0 | 0 | 0 | 0 | 0 | 0 |
| 214 | WT | wt/*aco1a* | 0 | 0 | 0 | 0 | 0 | 0 | 0 | 0 | 0 | 0 |
| 215 | *aco1a* | *aco1a/aco1a* | **2,5** | **3** | **0,5** | **1,5** | **3** | **2,5** | **2,8** | **3** | **3** | **2,8** |
| 216 | *aco1a* | *aco1a/aco1a* | **1,5** | **1** | **1** | **1** | **1,5** | **3** | **3** | **2,8** | **3** | **2,8** |
| 217 | *aco1a* | *aco1a/aco1a* | **3** | **2** | **3** | **3** | **3** | **3** | **3** | **3** | **3** | **3** |
| 218 | WT | wt/*aco1a* | 0 | 0 | 0 | 0 | 0 | 0 | 0 | 0 | 0 | 0 |
| 219 | WT | wt/*aco1a* | 0 | 0 | 0 | 0 | 0 | 0 | 0 | 0 | 0 | 0 |
| 220 | *aco1a* | *aco1a/aco1a* | 3 | 3 | 3 | 3 | 3 | 3 | 3 | 3 | 3 | 3 |
| 221 | WT | wt/*aco1a* | 0 | 0 | 0 | 0 | 0 | 0 | 0 | 0 | 0 | 0 |
| 222 | WT | wt/wt | 0 | 0 | 0 | 0 | 0 | 0 | 0 | 0 | 0 | 0 |
| 223 | WT | wt/*aco1a* | 0 | 0 | 0 | 0 | 0 | 0 | 0 | 0 | 0 | 0 |
| 224 | WT | wt/wt | 0 | 0 | 0 | 0 | 0 | 0 | 0 | 0 | 0 | 0 |
| 225 | WT | wt/*aco1a* | 0 | 0 | 0 | 0 | 0 | 0 | 0 | 0 | 0 | 0 |
| 226 | WT | wt/*aco1a* | 0 | 0 | 0 | 0 | 0 | 0 | 0 | 0 | 0 | 0 |
| 227 | *aco1a* | *aco1a/aco1a* | **3** | **1,5** | **2** | **2,5** | **1,5** | **3** | **1,8** | **2** | **0,3** | **2,8** |
| 228 | WT | wt/*aco1a* | 0 | 0 | 0 | 0 | 0 | 0 | 0 | 0 | 0 | 0 |
| 229 | WT | wt/wt | 0 | 0 | 0 | 0 | 0 | 0 | 0 | 0 | 0 | 0 |
| 230 | WT | wt/*aco1a* | 0 | 0 | 0 | 0 | 0 | 0 | 0 | 0 | 0 | 0 |
| 231 | WT | wt/*aco1a* | 0 | 0 | 0 | Determinate | | | | | | |
| 232 | WT | wt/*aco1a* | 0 | 0 | 0 | 0 | 0 | 0 | 0 | 0 | 0 | 0 |
| 233 | WT | wt/wt | 0 | 0 | 0 | 0 | 0 | 0 | 0 | 0 | 0 | 0 |
| 234 | WT | wt/*aco1a* | 0 | 0 | 0 | 0 | 0 | 0 | 0 | 0 | 0 | 0 |
| 235 | *aco1a* | *aco1a/aco1a* | **0** | **1** | **0,5** | **0,8** | **0,8** | **3** | **3** | **1,8** | **3** | **3** |
| 236 | *aco1a* | *aco1a/aco1a* | **0,5** | **1** | **1** | **1** | **1** | **2** | **3** | **1,8** | **2,5** | **2,3** |
| 237 | WT | wt/*aco1a* | 0 | 0 | 0 | 0 | 0 | 0 | 0 | 0 | 0 | 0 |
| 238 | WT | wt/wt | 0 | 0 | 0 | 0 | 0 | 0 | 0 | 0 | 0 | 0 |
| 239 | WT | wt/*aco1a* | 0 | 0 | 0 | 0 | 0 | 0 | 0 | 0 | 0 | 0 |
| 240 | WT | wt/*aco1a* | 0 | 0 | 0 | 0 | 0 | 0 | 0 | 0 | 0 | 0 |
| 241 | WT | wt/*aco1a* | 0 | 0 | 0 | 0 | 0 | 0 | 0 | 0 | 0 | 0 |
| 242 | WT | wt/wt | 0 | 0 | 0 | 0 | 0 | 0 | 0 | 0 | 0 | 0 |
| 243 | WT | wt/wt | 0 | 0 | 0 | 0 | 0 | 0 | 0 | 0 | 0 | 0 |
| 244 | *aco1a* | *aco1a/aco1a* | **3** | **2** | **1** | **2,5** | **2** | **3** | **3** | **3** | **3** | **3** |
| 245 | WT | wt/wt | 0 | 0 | 0 | 0 | 0 | 0 | 0 | 0 | 0 | 0 |
| 246 | WT | wt/*aco1a* | 0 | 0 | 0 | 0 | 0 | 0 | 0 | 0 | 0 | 0 |
| 247 | WT | wt/*aco1a* | 0 | 0 | 0 | 0 | 0 | 0 | 0 | 0 | 0 | 0 |
| 248 | WT | wt/*aco1a* | 0 | 0 | 0 | 0 | 0 | 0 | 0 | 0 | 0 | 0 |
| 249 | WT | wt/*aco1a* | 0 | 0 | 0 | 0 | 0 | 0 | 0 | 0 | 0 | 0 |
| 250 | *aco1a* | *aco1a/aco1a* | **3** | **1** | **1** | **2,5** | **1,8** | **1,8** | **2** | **1,8** | **1,8** | **2,8** |
| 251 | WT | wt/*aco1a* | 0 | 0 | 0 | 0 | 0 | 0 | 0 | 0 | 0 | 0 |
| 252 | WT | wt/*aco1a* | 0 | 0 | 0 | 0 | 0 | 0 | 0 | 0 | 0 | 0 |
| 253 | WT | wt/wt | 0 | 0 | 0 | 0 | 0 | 0 | 0 | 0 | 0 | 0 |
| 254 | WT | wt/wt | 0 | 0 | 0 | 0 | 0 | 0 | 0 | 0 | 0 | 0 |
| 255 | WT | wt/wt | 0 | 0 | 0 | 0 | 0 | 0 | 0 | 0 | 0 | 0 |
| 256 | WT | wt/*aco1a* | 0 | 0 | 0 | 0 | 0 | 0 | 0 | 0 | 0 | 0 |
| 257 | WT | wt/*aco1a* | 0 | 0 | 0 | 0 | 0 | 0 | 0 | 0 | 0 | 0 |
| 258 | WT | wt/*aco1a* | 0 | 0 | 0 | 0 | 0 | 0 | 0 | 0 | 0 | 0 |
| 259 | *aco1a* | *aco1a/aco1a* | **2** | **3** | **2,5** | **0,5** | **1** | **2,8** | **3** | **2,8** | **3** | **2,8** |
| 260 | *aco1a* | *aco1a/aco1a* | **3** | **3** | **0,5** | **1** | **1** | **2** | **1** | **3** | **3** | **3** |
| 261 | WT | wt/wt | 0 | 0 | 0 | 0 | 0 | 0 | 0 | 0 | 0 | 0 |
| 262 | WT | wt/wt | 0 | 0 | 0 | 0 | 0 | 0 | 0 | 0 | 0 | 0 |
| 263 | WT | wt/*aco1a* | 0 | 0 | 0 | 0 | 0 | 0 | 0 | 0 | 0 | 0 |
| 264 | WT | wt/*aco1a* | 0 | 0 | 0 | 0 | 0 | 0 | 0 | 0 | 0 | 0 |
| 265 | WT | wt/*aco1a* | 0 | 0 | 0 | 0 | 0 | 0 | 0 | 0 | 0 | 0 |
| 266 | WT | wt/wt | 0 | 0 | 0 | 0 | 0 | 0 | 0 | 0 | 0 | 0 |
| 267 | WT | wt/*aco1a* | 0 | 0 | 0 | 0 | 0 | 0 | 0 | 0 | 0 | 0 |
| 268 | WT | wt/*aco1a* | 0 | 0 | 0 | 0 | 0 | 0 | 0 | 0 | 0 | 0 |
| 269 | *aco1a* | *aco1a/aco1a* | **2,5** | **3** | **1,5** | **2** | **2,5** | **3** | **1,5** | **1** | **2,8** | **3** |
| 270 | WT | wt/*aco1a* | 0 | 0 | 0 | 0 | 0 | 0 | 0 | 0 | 0 | 0 |
| 271 | WT | wt/*aco1a* | 0 | 0 | 0 | 0 | 0 | 0 | 0 | 0 | 0 | 0 |
| 272 | *aco1a* | *aco1a/aco1a* | **1,5** | **2** | **1** | **2,5** | **3** | **1,5** | **2,8** | **3** | **3** | **2** |
| 273 | *aco1a* | *aco1a/aco1a* | **3** | **2,5** | **2,5** | **2** | **1** | **1** | **1,5** | **2** | **2,5** | **1,8** |
| 274 | *aco1a* | *aco1a/aco1a* | **3** | **3** | **1** | **2** | **1,5** | **1** | **3** | **2,8** | **3** | **2,8** |
| 275 | WT | wt/*aco1a* | 0 | 0 | 0 | 0 | 0 | 0 | 0 | 0 | 0 | 0 |
| 276 | WT | wt/*aco1a* | 0 | 0 | 0 | 0 | 0 | 0 | 0 | 0 | 0 | 0 |
| 277 | WT | wt/wt | 0 | 0 | 0 | 0 | 0 | 0 | 0 | 0 | 0 | 0 |
| 278 | WT | wt/*aco1a* | 0 | 0 | 0 | 0 | 0 | 0 | 0 | 0 | 0 | 0 |
| 279 | WT | wt/wt | 0 | 0 | 0 | 0 | 0 | 0 | 0 | 0 | 0 | 0 |
| 280 | WT | wt/*aco1a* | 0 | 0 | 0 | 0 | 0 | 0 | 0 | 0 | 0 | 0 |
| 281 | WT | wt/*aco1a* | 0 | 0 | 0 | 0 | 0 | 0 | 0 | 0 | 0 | 0 |
| 282 | WT | wt/wt | 0 | 0 | 0 | 0 | 0 | 0 | 0 | 0 | 0 | 0 |
| 283 | WT | wt/wt | 0 | 0 | 0 | 0 | 0 | 0 | 0 | 0 | 0 | 0 |
| 284 | WT | wt/*aco1a* | 0 | 0 | 0 | 0 | 0 | 0 | 0 | 0 | 0 | 0 |
| 285 | WT | wt/*aco1a* | 0 | 0 | 0 | 0 | 0 | 0 | 0 | 0 | 0 | 0 |
| 286 | WT | wt/wt | 0 | 0 | 0 | 0 | 0 | 0 | 0 | 0 | 0 | 0 |
| 287 | WT | wt/*aco1a* | 0 | 0 | 0 | 0 | 0 | 0 | 0 | 0 | 0 | 0 |
| 288 | WT | wt/*aco1a* | 0 | 0 | 0 | 0 | 0 | 0 | 0 | 0 | 0 | 0 |
| 289 | *aco1a* | *aco1a/aco1a* | **1,5** | **3** | **3** | **2,8** | **3** | **3** | **3** | **3** | **3** | **3** |
| 290 | *aco1a* | *aco1a/aco1a* | **3** | **3** | **3** | **3** | **3** | **3** | **2,5** | **2,8** | **3** | **3** |
| 291 | WT | wt/*aco1a* | 0 | 0 | 0 | 0 | 0 | 0 | 0 | 0 | 0 | 0 |
| 292 | WT | wt/wt | 0 | 0 | 0 | 0 | 0 | 0 | 0 | 0 | 0 | 0 |
| 293 | WT | wt/*aco1a* | 0 | 0 | 0 | 0 | 0 | 0 | 0 | 0 | 0 | 0 |
| 294 | *aco1a* | *aco1a/aco1a* | **1** | **0,5** | **2,5** | **1,5** | **3** | **3** | **3** | **3** | **3** | **2** |
| 295 | *aco1a* | *aco1a/aco1a* | **0** | **0** | **0,5** | **1** | **1** | **0,5** | **0,5** | **3** | **3** | **3** |
| 296 | WT | wt/*aco1a* | 0 | 0 | 0 | 0 | 0 | 0 | 0 | 0 | 0 | 0 |
| 297 | WT | wt/*aco1a* | 0 | 0 | 0 | 0 | 0 | 0 | 0 | 0 | 0 | 0 |
| 298 | WT | wt/wt | 0 | 0 | 0 | 0 | 0 | 0 | 0 | 0 | 0 | 0 |
| 299 | WT | wt/wt | 0 | 0 | 0 | 0 | 0 | 0 | 0 | 0 | 0 | 0 |
| 300 | WT | wt/wt | 0 | 0 | 0 | 0 | 0 | 0 | 0 | 0 | 0 | 0 |

| **Table S5**. EMS mutations in chromosome 4 of *aco1a* mutant line. | | | | | | |
| --- | --- | --- | --- | --- | --- | --- |
| **Chr** | **Position** | **Ref** | **Alt** | **Gene ID** | **Impact** | **Functional Annotation** |
| 4 | 3,982,041 | G | A | Cp4.1LG04g07570 | Intron | ATP-dependent Clp protease proteolytic subunit |
| 4 | 5,341,391 | C | T | Cp4.1LG04g06230 | 3´UTR | Shikimate kinase |
| 4 | 7,603,991 | C | T | Intergenic | intergenic | between Cp4.1LG04g02740 and Cp4.1LG04g02810 |
| 4 | 7,715,975 | C | T | Cp4.1LG04g02610 | Exon, P5L | 1-aminocyclopropane-1-carboxylate oxidase 1 (ACO1) |

The SNPs were genotyped in a BC_2_S_1_ population segregating for the *aco1a* phenotype. Only the mutation C>T in Cp4.1LG04g02610 co-segregated with the mutant phenotype in a total of 300 plant analyzed.
